# Antioxidants rescue murine mesangial cells from docosahexaenoic acid-induced ferroptosis

**DOI:** 10.1186/s40348-025-00215-y

**Published:** 2026-01-05

**Authors:** Leon Saschin, Anna Kowalewski, Gregor Fink, Jenny Voggel, Maria Wohlfarth, Lea Quell, Jörg Dötsch, Miguel A. Alejandre Alcázar, Kai-Dietrich Nüsken, Eva Nüsken

**Affiliations:** 1 Department of Pediatric and Adolescent Medicine, Faculty of Medicine, University Hospital Cologne, University of Cologne, Kerpener Str. 62, 50937 Cologne, Germany; 2 Department of Pediatric and Adolescent Medicine, Translational Experimental Pediatrics, Experimental Pulmonology, Faculty of Medicine and University Hospital Cologne, University of Cologne, Cologne, Germany; 3 Cologne Excellence Cluster on Cellular Stress Responses in Aging-Associated Diseases (CECAD) and Center for Molecular Medicine Cologne (CMMC), University of Cologne, Cologne, Germany; 4 Institute for Lung Health, University of Giessen and Marburg Lung Center (UGMLC), Member of the German Center for Lung Research (DZL), Gießen, Germany

**Keywords:** Ferroptosis, Omega-3 polyunsaturated fatty acids, Kidney health, Antioxidants, Oxidative stress

## Abstract

**Background:**

Omega-3 polyunsaturated fatty acids (n-3 PUFAs) are associated with anti-inflammatory effects. However, few studies have investigated their molecular effects in the kidney. We have previously shown that intrauterine growth restriction (IUGR) can lead to aggravation of mesangioproliferative glomerulonephritis and that n-3 PUFAs can attenuate long-term effects of IUGR by reversing pro-inflammatory molecular signatures in kidney cortex.

**Results:**

The original aim of the study was to investigate the potential protective mechanisms of docosahexaenoic acid (DHA) on murine mesangial cells. However, stimulation with DHA alone led to reduced cell viability and ultimately cell death. Proteome analysis revealed significant regulation of ferroptosis-associated proteins. Increased expression of the pro-ferroptotic protein HMOX1 and decreased expression of the pro-ferroptotic proteins TFRC and ACSL4 could indicate the onset of self-protection mechanisms in ferroptosis that is already underway. Interestingly, treatment with the ferroptosis inhibitor ferrostatin-1 maintained cellular metabolic activity and prevented cell death, further supporting a role of ferroptosis in DHA-induced cytotoxicity. Consistently, DHA-treated cells exhibited pronounced lipid peroxidation while showing no relevant activation of apoptosis. Simultaneous treatment with DHA and an antioxidant cocktail significantly attenuated cell death and induced upregulation of several key anti-ferroptotic proteins, including TXNRD1 and GPX4, while pro-ferroptotic proteins such as TFRC and ASCL4 were further reduced.

**Conclusion:**

Our results provide evidence that DHA-treatment alone may have detrimental effects in susceptible cells, which could partially explain inconsistent results of clinical studies. This emphasizes the importance of a balance between pro- and anti-ferroptotic mechanisms in therapeutic strategies using n-3 PUFAs to promote kidney health.

**Supplementary Information:**

The online version contains supplementary material available at 10.1186/s40348-025-00215-y.

## Background

Omega-3 polyunsaturated fatty acids (n-3 PUFAs) are associated with multiple health benefits. Despite extensive research on cardiovascular or metabolic benefits, few studies have investigated the molecular effects of n-3 PUFAs on kidney health. Although some studies suggest positive effects on renal outcome [1–4], large and randomized clinical trials focusing on kidney health as the primary endpoint have not been conducted so far. Most evidence comes from animal studies.

Thus, fish oil-fed Milan Normotensive Rats (MNR), which naturally develop proteinuria, showed significantly lower albuminuria, lower cholesterol and triglyceride levels, and less interstitial damage compared to safflower oil or lard [5]. In another in vivo study, feeding salmon oil appeared to have an overall beneficial effect on kidney histology compared to feeding beef tallow in partially nephrectomized rats, while slowing the progression of kidney failure [6]. Similarly, rats with chronic kidney failure exhibited slower disease progression when fed a diet rich in n-3 PUFAs [7]. Single studies have studied a combination of n-3 PUFAs with specific antioxidants such as vitamin E (tocopherol). Thus, in a 5/6 renal ablation model in rats, the combination therapy helped to preserve the glomerular filtration rate and treated animals had less albuminuria in comparison to a regular laboratory diet [8]. In addition, dyslipidemia was prevented in the n-3 PUFA group [6, 8]. However, although beneficial effects of n-3 PUFA supplementation on kidney health are well documented in these studies, the underlying molecular mechanisms remain elusive.

The original aim of this study was to elucidate the protective molecular effects of DHA treatment at the cellular level. Since our group has shown that intrauterine growth restriction can lead to a more severe course of mesangioproliferative glomerulonephritis [9] and that n-3 PUFA enriched diet during postnatal kidney development has the potential to attenuate the long-term effects of unfavorable intrauterine conditions on kidney health by reversing pro-inflammatory signatures in kidney cortex tissue [10], we chose mesangial cells as the experimental setting. Mesangial cells, which make up about one third of glomerular cells, are a biologically and pathophysiologically highly relevant target for scientific investigations due to their central role in glomerular homeostasis. The cells are highly involved in the onset and development of glomerular damage, including various forms of glomerulonephritis, and play a key role in fibrotic changes [11]. The cells actively contribute to fibrotic remodeling and inflammation by interacting in dynamic, tri-directional manner with neighboring glomerular cells, such as endothelial cells and podocytes, by releasing cytokines and chemokines that influence each other [12]. Their capacity to modulate inflammatory signaling pathways positions them as key effectors in the pathogenesis of glomerular injury [11]. Due to their regulatory role, mesangial cells are therefore also a promising but so far rather neglected therapeutic target for interventions aimed at reducing pro-inflammatory pathways and glomerular diseases. When DHA treatment of mesangial cells unexpectedly resulted in a decrease in viability and increased cell death rate, we expanded the study and wanted to establish a treatment protocol that would allow further investigation of DHA as a therapeutic agent in mesangial cells. Since docosahexaenoic acid (DHA), a major n-3 PUFA, is known for its susceptibility to peroxidation and DHA has already been used together with antioxidants for this purpose in some studies [13, 14], we focused on co-treatment with antioxidants to rescue the cells. Finally, we performed proteomics to elucidate the molecular mechanisms leading to DHA-induced cell death and to understand the rescuing effect of antioxidants.

## Methods

### Cell lines and culturing conditions

Murine mesangial SV40 MES 13 cells were obtained from the American Type Culture Collection (ATCC; Rockville, MD, USA). The cells are originally derived from transgenic mice and exhibit biochemical and morphological features of normal mesangial cells in culture. The cells were maintained in DMEM: F12 (3:1) medium containing 4.5 g/l glucose, which was supplemented with 5% fetal calf serum, 1% penicillin/streptomycin, HEPES (1 M), MgCl2 and L-glutamine. In order to create a physiologically relevant environment in which fatty acids are supplied bound to serum proteins, the experiments were conducted in serum-rich medium without depletion. To ensure consistency, all media were prepared from a single serum batch. Cells routinely were grown to confluence in a humidified 37 °C/5% CO_2_ incubator. For stimulation, 10.5 × 10^5^ cells were seeded in a petri dish and let grow for 16 h. The cells were either stimulated in serum-rich medium supplemented with DHA, oxidized DHA (oxDHA) (both 25 µM; complexed to 0.25% BSA as a carrier), or with a commercially available antioxidant cocktail (1:1000, v/v; Sigma-Aldrich; St. Louis, MO, USA) in different combinations for 24 h. The selected concentration and stimulation time were optimized in preliminary tests and correspond to the protocols specified in the literature [15, 16]. The final study design reliably led to a measurable biological effect without causing overwhelming cell death, thereby maintaining pathophysiological relevance. Oxidized DHA was produced in-house by auto-oxidation. Briefly, a DHA solution was incubated for 24 h at 37 °C in a standard cell culture incubator to promote radical-mediated lipid peroxidation *via* continuous exposure to atmospheric oxygen and heat. Appropriate controls were carried along. All treatments, if not stated otherwise, were performed in five independent experiments, constituting five biological replicates (*n* = 5).

### Protein extraction and in-solution digestion

Cell pellets were lysed in 8 M urea/50 mM Tetraethylammonium Bromide (TEAB) containing protease inhibitors. Following centrifugation (20,000 *x* g, 15 min, 4 °C), protein concentrations were quantified using the Pierce 660 nm Protein Assay (Thermo Scientific) according to manufacturer’s instruction. Proteins were reduced with 5 mM Dithiothreitol (DTT) (1 h, 25 °C) and alkylated with 40 mM chloroacetamide (CAA) (30 min, RT, in the dark). Digestion was performed with Lysyl endopeptidase (LysC) (1:75, v/v, 2 h, 25 °C), followed by dilution with TEAB to < 2 M urea and overnight digestion with trypsin (1:75, v/v, 25 °C). The reaction was quenched with 1% formic acid. Peptides were purified using C18 StageTips, washed with 0.1% formic acid, and eluted with 80% acetonitrile/0.1% formic acid. Samples were completely dried and stored at 4 °C until LC-MS/MS analysis (Liquid Chromatography-Tandem Mass Spectrometry).

### Mass spectrometry (as performed by the proteomics facility of the cologne excellence cluster for aging and aging-associated diseases (CECAD))

Mass spectrometry involved an Orbitrap Exploris 480 mass spectrometer (Thermo Scientific, Waltham, MA, USA) equipped with a FAIMSpro differential ion mobility device (Thermo Scientific, Waltham, MA, USA) and coupled to an UltiMate 3000 RSLC system (Thermo Scientific, Waltham, MA, USA). Analysis of the samples was performed according to Nüsken et al. 2019 [17] using data-independent acquisition mode (DIA-NN) 1.8.1 [18].

### Proteomics data analysis (as performed by the proteomics facility of the CECAD Cologne)

Data were analyzed using DIA-NN 1.8.1 [18]. A spectral library was generated based on the canonical SwissProt mouse database (UP589, downloaded 18.06.2022). Data were mapped to this library, and results were filtered for a library q-value and global q-value ≤ 0.05 with a minimum of two unique peptides per protein using R (v4.1.3). LFQ values were calculated with the DIA-NN R package, and statistical analyses were conducted in Perseus 1.6.15. One-way ANOVA and two-sample t-tests were used, with a significance threshold of *P* < 0.05. Proteins were considered significantly differentially expressed if they met the significance criterion and had a log2 fold change >|0.58| (fold change ≥ 1.5). Only proteins quantified in at least three of five replicates within test groups were included. Key metrics from the results included the number of valid values, -Log10 p-values (from Student’s t-tests), and log2 fold changes, which were used to identify significantly regulated proteins for further analysis.

### Further data processing

Subsequently, we used String database analysis (www.string-db.com, version 11.8.1) to identify molecular interactions and functional enrichments concerning biological processes or Uniprot Keywords (false discovery rate FDR < 0.01) of all significantly (*p* < 0.05) and relevantly (fold change, fc ≥ 1.5 or ≤ −1.5) altered proteins. Statistically significant proteins (*p* < 0.05) with a fold change (fc) of ≥ 1.5 *or* ≤ −1.5 relative to the control group were subjected to further analysis to explore molecular interactions and functional enrichment. To ensure robust comparison, only proteins with reliable quantifications in at least three out of five independent runs were included in the final analysis.

### Incucyte live-cell imaging and cell death measurement

Live-cell imaging and cell death measurements were performed using the Incucyte S3 (Sartorius) at 37 °C and 5% CO₂. For this, cells were seeded in 24-well plates (30,000 cells/well) and treated with the respective stimuli. Moreover, cells were stained with the fluorescent dye DRAQ7 (1:2000), which selectively stains double-stranded DNA in dead and permeabilized cells and incubated for 24 h in total. During incubation, four images per well were acquired every hour and analyzed using the Incucyte basic analysis software module. Cell death was measured by the incorporation of DRAQ7 into dead cells, which stained the cells red. The total cell count was determined by automated analysis of phase-contrast images using the Incucyte’s integrated cell-by-cell counting algorithm. This software creates a mask based on phase-contrast images to identify and count individual cell in each well, providing a reliable measure of the total cell population at each time point. The number of DRAQ7-positive (dying/dead) cells was then normalized to this total cell count to calculate the percentage of dead cells. As an additional control, the total cell count for each condition was also normalized to the cell count at the experiment’s starting point (time-point zero) to account for any potential effects on cell proliferation. Each biological replicate (*n* = 5) was measured in technical duplicates, which were then averaged to a single value.

### Measurement of the metabolic activity and viability

Metabolic activity, as an indicator of cell viability, was assessed using the MTT assay. Cells were seeded in 96-well plates at a density of 25,000 cells per well and incubated for 24 h at 37 °C and 5% CO₂ to allow adherence. Following respective stimulations for 24 h, 100 µL of MTT solution (0.5% w/v, equivalent to 5 mg/mL) was added to each well. The plates were then incubated for 4 h to facilitate the formation of purple formazan crystals. To stop the reaction, 50 µL of a 1:1 (v/v) water-NTT solution containing 10% SDS was added to each well. The wells were thoroughly mixed to ensure complete dissolution of the formazan crystals. Absorbance was measured at 570 nm, with 655 nm as the reference wavelength, using a TECAN Spark microplate reader both immediately and after 20 min of incubation. The recorded absorbance values directly correlate with the metabolic activity of the cells. For each biological replicate (*n* = 5), six technical replicates were measured and averaged to obtain a single value per biological subject.

### Immunoblotting via Western blotting technique

Protein samples, previously isolated and prepared (reduced and alkylated) for proteomic analysis, were utilized. Protein concentration was determined using the Pierce BCA Protein Assay Kit (Thermo Fisher Scientific, Waltham, MA, USA) according to manufacturer’s instructions and measured with the TECAN Spark microplate reader. For immunoblotting, 20 µg of protein per sample were loaded on a 15% polyacrylamide gel, separated by sodium dodecyl sulfate polyacrylamide gel electrophoresis (SDS-PAGE), and transferred on a nitrocellulose membrane for 120 min at 1.3 mA/cm^2^ using semi-dry blotting. Membranes were blocked with 5% milk and 2% BSA in Tris-buffered saline containing 0.05% Tween 20 (TBST) and subsequently incubated overnight with the following antibodies: monoclonal rabbit anti-FTH1 (Cell Signaling Technology, #4393; 1:1000), monoclonal rabbit anti-GPX4 (abcam, ab125066; 1:1000), polyclonal rabbit anti-Caspase-3 (Cell Signaling Technology, #9662; 1:1000), polyclonal rabbit anti-cleaved Caspase-3 (Cell Signaling Technology, #9661; 1:500), whereas monoclonal mouse anti-α-Tubulin (Sigma-Aldrich, #T9026; 1:3000) served as a loading control. Anti-mouse IgG, HRP-linked (Cell Signaling Technology, #7076; 1:3000/1:4000), and anti-rabbit, HRP-linked (Cell Signaling Technology, #7074; 1:1000/1:2000/1:4000) were used as secondary antibodies. Protein bands were developed using an enhanced chemiluminescence (ECL) substrate and the ChemiDoc XRS + Imaging System. Densiometric analysis of protein bands was performed using Bio-Rad ImageLab software (Version 5.2.1, Bio-Rad, Munich, Germany). Band intensities from samples were normalized for loading using the α-Tubulin band from the same sample.

### Live-cell imaging of lipid peroxidation

Lipid peroxidation was assessed in live cells using the fluorescent probe Bodipy^581/591^ C-11 (Thermo Scientific, Waltham, MA, USA). Briefly, 25,000 cells per well were seeded in 8-well Lumox chamber slides (Sarstedt, Nümbrecht, Germany) and allowed to adhere for 16–18 h. Cells were then subjected to the experimental treatments as described elsewhere with 25 µM DHA, 25 µM DHA + AO, or the control medium. As a positive control for lipid peroxidation, cells were exposed to UV light for 5 min prior to staining.

A 1 mg/mL stock solution of Bodipy^581/591^ C-11 was prepared in DMSO and further diluted in cell culture medium to a final working concentration of 5 µM. Cells were incubated with the working solution for 30 min at 37 °C and 5% CO2 in the dark. Following incubation, cells were gently washed three times with PBS and immediately imaged in PBS.

The Bodipy^581/591^ C-11 probe exhibits a fluorescent shift upon oxidation: it emits red fluorescence in its reduced state and shifts to green upon oxidation. Therefore, an increase in green fluorescence indicated lipid peroxidation. Due to the qualitative nature of this assay, representative images are presented without quantification. For improved visualization of individual channels, brightness was uniformly increased (oxidized Bodipy^581/591^ C-11: 15%; reduced Bodipy^581/591^ C-11: 30%) without contrast adjustment. The merged image represents unmodified channel overlays.

### Statistics

Statistical details of the individual experiments are described in the corresponding figure and table legends. GraphPad Prism 9 was used for statistical analyses. In general, statistical testing was performed according to recommendations of the software.

## Results

### Stimulation of murine mesangial cells with DHA promotes cell death

**Fig. 1:**
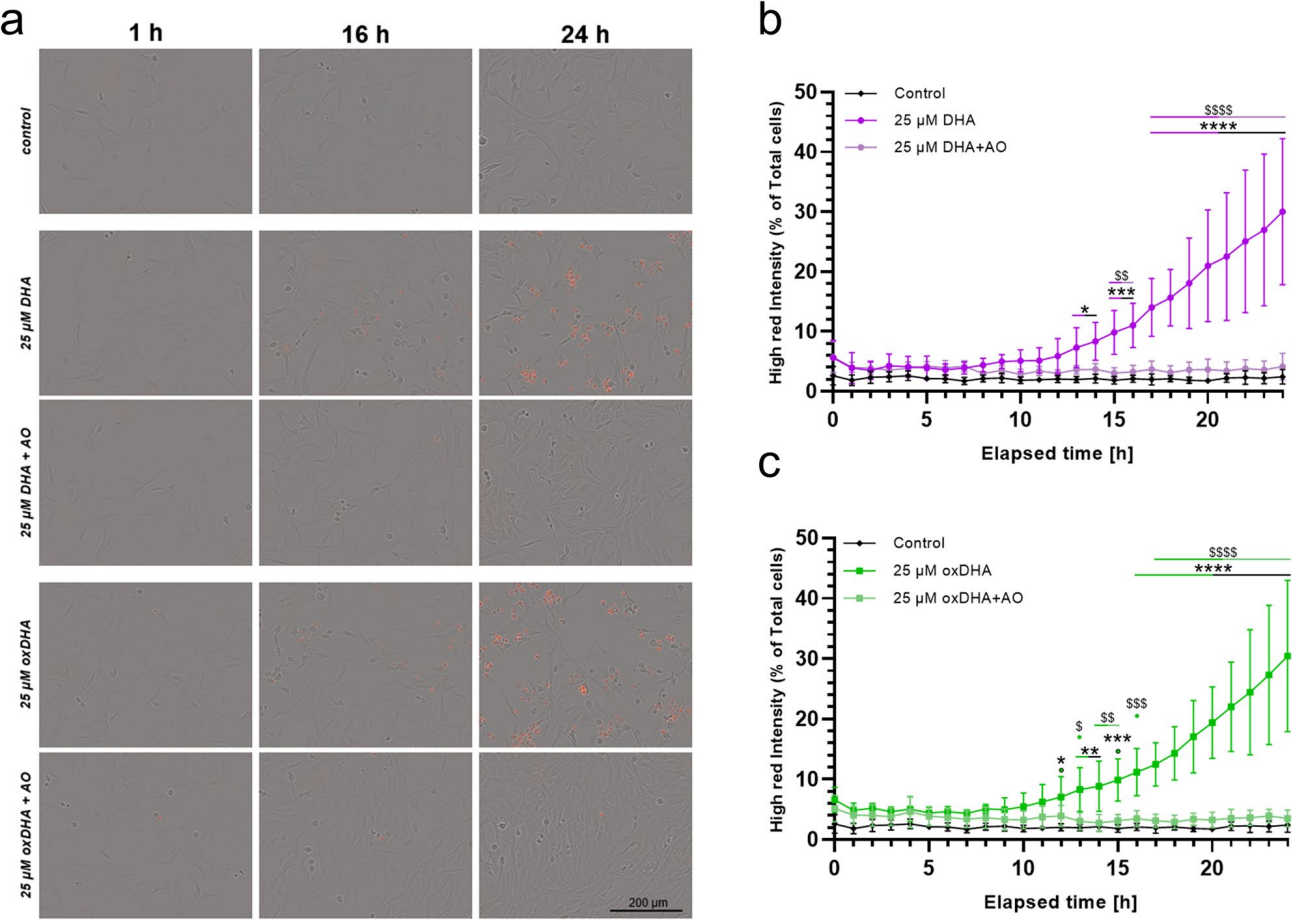
Impact of DHA and antioxidants on cell viability of murine mesangial cells. **A **exemplary images after 1, 16, and 24 h. Red coloring indicates cell death. B + C. quantification of cell death, based on red staining of dead cells (high red intensity). **B** Comparison between control, DHA, and DHA + AO stimulated groups. **C** Comparison between control, oxDHA, and oxDHA + AO stimulated groups. Experiment performed with the Incucyte. Results are depicted as means (*n* = 5, biological replicates) ± SD. Two-way ANOVA and post-hoc Tukey’s test for multiple comparisons was conducted. Significance is depicted with asterisks (control vs. DHA) or Dollar signs (DHA vs. DHA + AO). Significance for oxDHA stimulated groups is depicted respectively. (*, *p* < 0.05; **, *p* < 0.01; ***, *p* < 0.001; ****, *p* < 0.0001). Each biological replicate was measured in technical duplicates

As mentioned above, DHA is an n-3 PUFA that is highly susceptible to oxidative degradation by free radicals due to its high degree of unsaturation. To investigate the effects DHA alone as well as oxidized DHA (oxDHA) alone on the induction of cell death in vitro, we performed live cell imaging experiments. Differences in cell viability over time were observed between cells treated either with 25 µM DHA or oxDHA and the control group. Both DHA (Fig. 1a and b) and oxDHA (Fig. 1a and c) treatments led to a significant increase in high red intensity, indicative of dead cell count, since the far-red fluorescent DNA intercalator DRAQ7 only stains nuclei in cells with compromised membranes. In the control groups, the percentage of high red intensity cells remained low and stable, averaging between 0% and 10% throughout the experiment. In contrast, cells exposed to 25 µM DHA displayed an increasing percentage of high red intensity cells over time beginning around the 10-hour mark, with a significant rise observed after ~ 13 h (DHA: 7.32 ± 3.34% vs. Control: 1.99 ± 0.52% dead cells; *p* = 0.0421; *n* = 5), as indicated by asterisks. By the end of the 24-hour period, the DHA-treated group approached nearly 30% cell death (Fig. 1a and b). Cells exposed to 25 µM oxDHA displayed a similar course after ~ 13 h (oxDHA: 8.31 ± 3.62% vs. Control: 1.99 ± 0.52% dead cells; *p* = 0.0044; *n* = 5) and 24 h (Fig. 1a and c).

### Co-stimulation with antioxidants rescues mesangial cells from DHA-induced cell death

In both DHA and oxDHA treatments, co-stimulation with an antioxidant cocktail (AO) effectively mitigated the rise in dead cell count (DHA, Fig. 1a and b; oxDHA, Fig. 1a and c). Both groups co-treated with AO exhibited a viability trend similar to the control group, with only a minimal non-significant increase in high red intensity over time. Statistical analysis revealed significant differences between the DHA/oxDHA-only and DHA/oxDHA + AO groups at multiple time points (Fig. 1b and c, noted by dollar signs ($)), with *p* < 0.01 and *p* < 0.0001, diverging significantly after ~ 14 h of stimulation. These results indicate that co-treatment with antioxidants mitigates the cytotoxic effects of both DHA and oxDHA, preserving cell viability over the experimental timeframe.

### Metabolic activity in murine mesangial cells is reduced after stimulation with DHA

To assess the viability of murine mesangial cells following stimulation with 25 µM DHA, with and without co-stimulation by antioxidants (AO), we conducted an MTT assay. Given that preliminary results showed no significant difference in cell survival between DHA- and oxidized DHA (oxDHA)-stimulated groups subsequent experiments omitted the oxDHA condition. This study included six treatment groups: four controls ((1) standard culture medium (Control); (4) carrier control (0.25% BSA); (5) antioxidant-only control; (6) cell-free negative control) and two experimental groups ((2) DHA 25 µM; (3) DHA 25 µM + AO). The detailed composition of the cell culture medium is described in the previous section.

First, there was no significant difference in metabolic activity between the control (1.00 ± 0.10), the carrier control (0.97 ± 0.10), and the AO-only control (0.93 ± 0.14), confirming that neither bovine serum albumin (BSA) nor antioxidants alone impact mesangial cell viability.

**Fig. 2:**
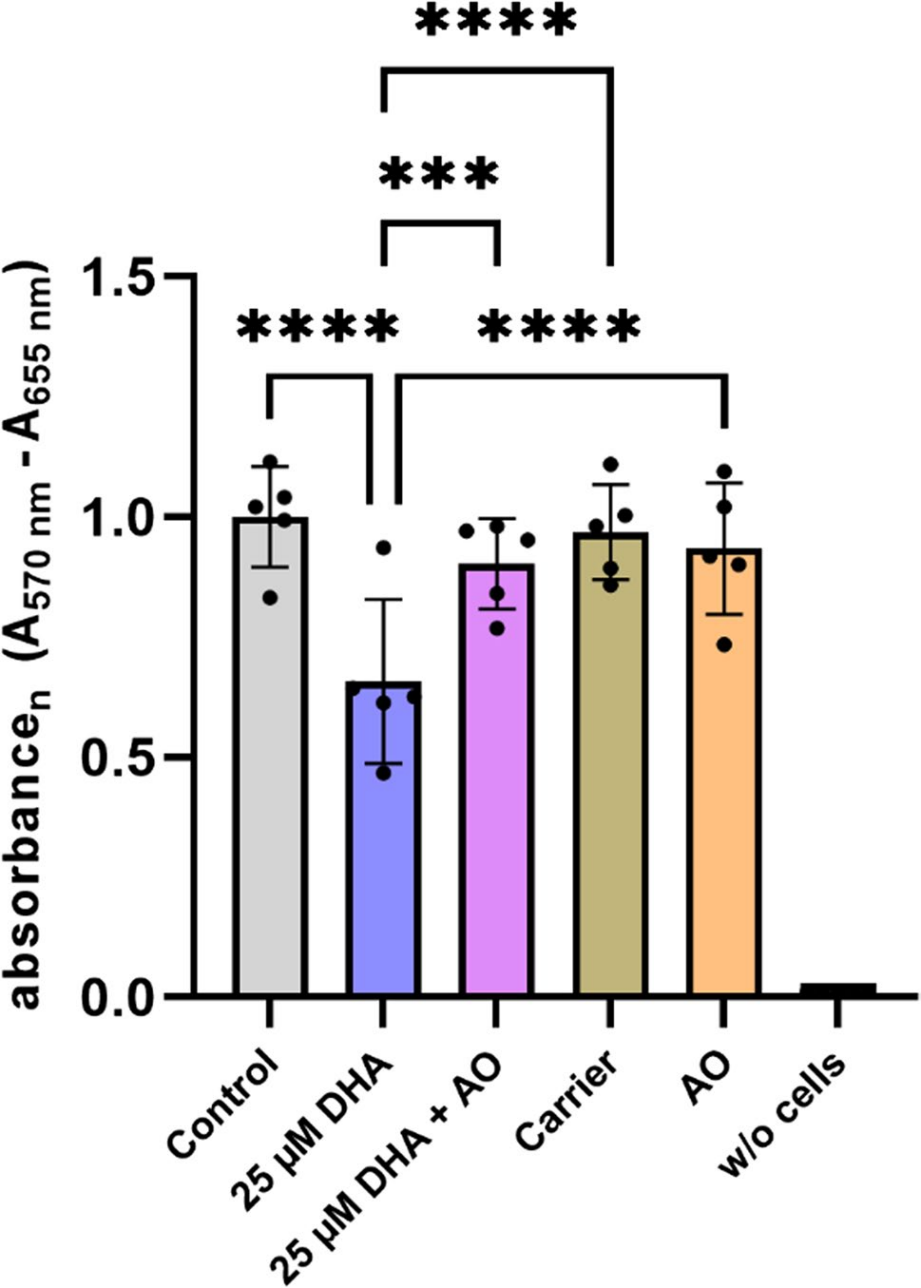
Impact of DHA and antioxidant treatment on metabolic activity. MTT assay results assessing metabolic activity under different treatment conditions. Depicted as relative absorbance values measured at A570nm – A655nm normalized to the control group Bars represent mean ± SD (*n* = 5, biological replicates). Statistical analysis was performed using two-way ANOVA and post-hoc Tukey’s test for multiple comparisons was conducted. Significant differences are indicated by asterisks (*, *p* < 0.05; **, *p* < 0.01; ***, *p* < 0.001; ****, *p* < 0.0001). Not all pairwise comparisons are shown. For each biological replicate, six technical replicates were measured and averaged

Second, stimulation with 25 µM DHA significantly decreased metabolic activity compared to the standard group (DHA: 0.66 ± 0.17 vs. Control: 1.00 ± 0.10; *p* < 0.0001; *n* = 5), as depicted in Fig. 2. This reduction highlights a negative impact on cell viability when mesangial cells are exposed to DHA.

In contrast, co-stimulation with DHA and antioxidants (DHA + AO) mitigated this reduction in metabolic activity, resulting in significantly higher viability compared to DHA alone (DHA + AO: 0.90 ± 0.09 vs. DHA: 0.66 ± 0.17; *p* < 0.001; *n* = 5). Furthermore, there was no statistically significant difference between the Control and the DHA + AO group (Control: 1.00 ± 0.10 vs. DHA + AO: 0.90 ± 0.09).

These results suggest that while DHA alone impairs cell viability, simultaneous stimulation with antioxidants effectively prevents this effect and maintains metabolic function (Fig. 2).

### Proteomic analysis of murine mesangial cells following DHA and antioxidant stimulation

To assess the molecular impact of docosahexaenoic acid (DHA) and antioxidants (AO) on mesangial cells in vitro, we conducted a comprehensive proteomic analysis following seven different stimulation conditions. An open, unbiased, and hypothesis-generating approach was employed to capture a broad range of molecular responses. Proteomic profiling was performed under these conditions: (1) control (only medium); (2) 25 µM DHA; (3) 25 µM DHA + AO; (4) 25 µM oxidized DHA (oxDHA); (5) 25 µM oxDHA + AO; (6) Carrier; and (7) AO.

In total, ninety-one proteins were significantly (*p* < 0.05) and substantially (fold change [fc] of ≥ 1.5 *or* ≤ −1.5) altered in murine mesangial cells following stimulation, compared to the control group. Specifically, 29 differentially expressed proteins were identified in the DHA-stimulated group (i.e., DHA vs. control, Table 2), 31 proteins in the DHA + AO group (i.e. DHA + AO vs. control, Table 3), 40 proteins in the oxDHA group (i.e., oxDHA vs. control, Supplementary Table 3), and 37 proteins in the oxDHA + AO group (i.e., oxDHA + AO vs. control, Supp. Table 4). Additionally, the Carrier (i.e., Carrier vs. control, Supp. Table 1) and AO (i.e., AO vs. control, Supp. Table 2) groups showed 7 and 5 differentially expressed proteins, respectively.

### Docosahexaenoic acid significantly and relevantly affects pathways of ferroptosis, fatty acid metabolism/degradation, and PPAR signaling pathway

Once differentially expressed proteins had been identified, pathway and network analyses were performed using the STRING database. The analysis within each treatment group revealed modulation of various KEGG pathways, as outlined in Table 1>. Notably, pathways related to Ferroptosis, Fatty Acid Metabolism, and PPAR Signaling were consistently regulated across all stimulation conditions. Furthermore, the pathway of Fatty Acid Degradation was modulated in the DHA, DHA + AO, and oxDHA + AO groups. Given the minimal differences in protein regulation between the DHA- and oxDHA-stimulated groups, the following presentation of specific proteins primarily focuses on the DHA-stimulated groups (see Tables 2 and 3).

**Table 1:**
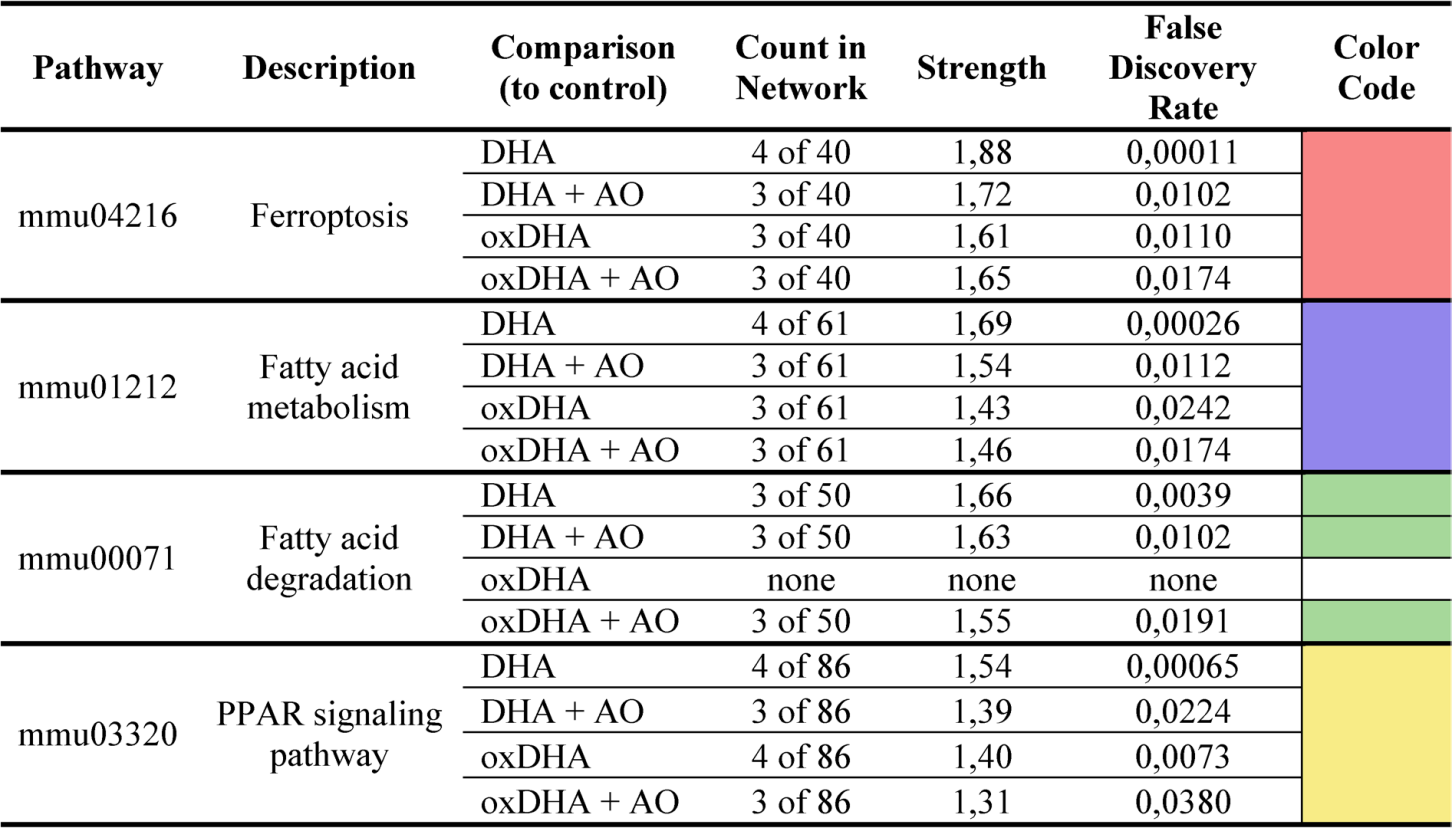
Relevant pathways identified through STRING (string.db) database

The columns display the KEGG pathway ID, pathway description, group comparison (relative to control), the count of proteins involved in each pathway network, enrichment strength, and the false discovery rate (FDR) for each pathway. Each pathway is color coded (ferroptosis, red; fatty acid metabolism, blueish; fatty acid degradation, green; PPAR signaling pathway, yellow). These pathways represent the only statistically relevant findings from the pathway analysis, indicating their potential role in mediating the cellular response to these treatments

**Table 2:**
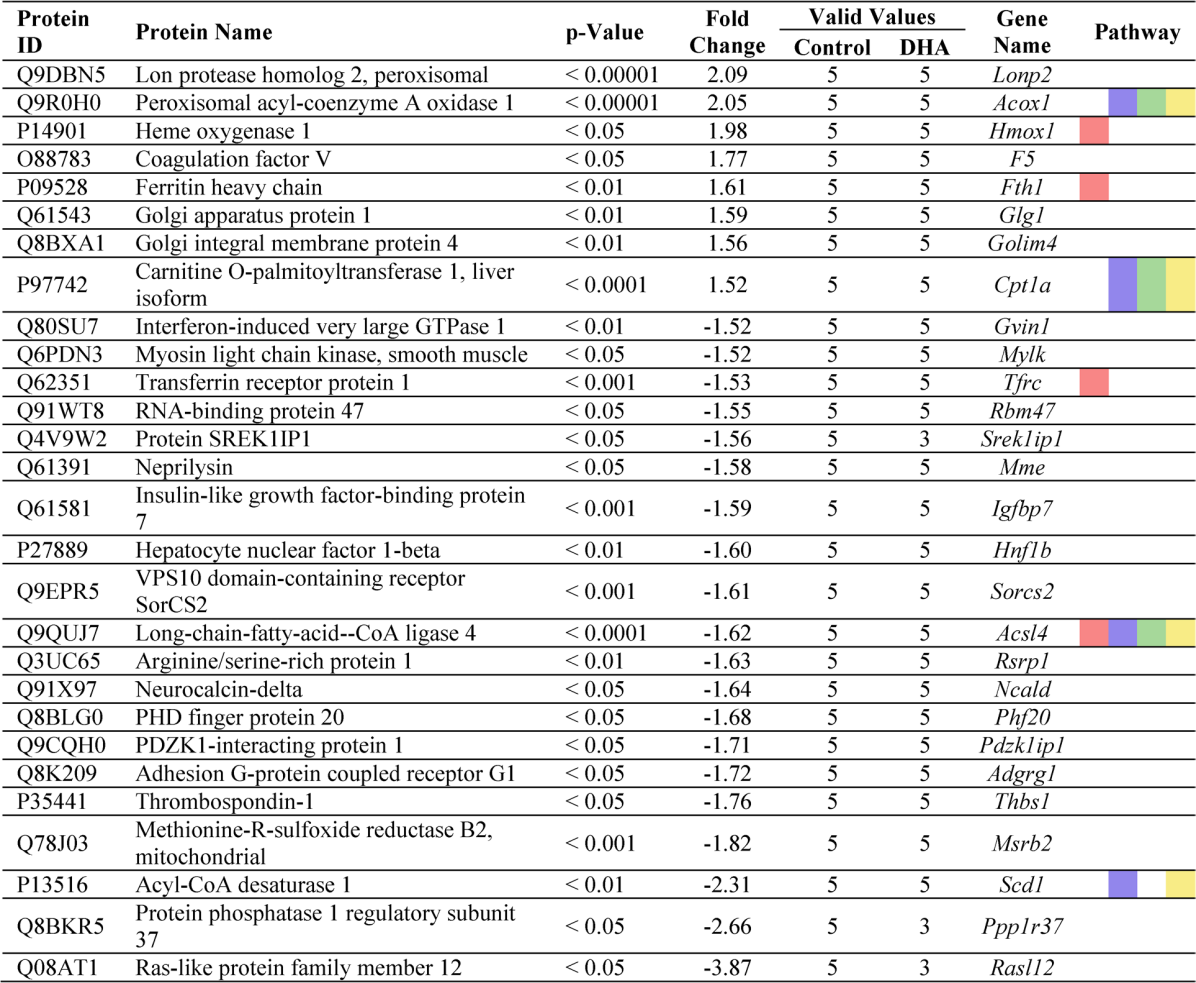
Differentially expression proteins in mesangial cells following DHA stimulation, based on the analysis of the cellular proteome

This table lists the proteins, which are differentially expressed between the DHA-stimulated cells and the control group, including their respective protein ID, name, p-value, fold-change, and gene name. The column “valid values” indicated the number of valid measurements for each protein, while only proteins with at least 3 valid measurements in both groups were included. The column “pathways” shows the relevant pathways associated with each protein, color-coded to match pathway categories presented in related analysis tables (Table 1). Proteins are sorted in descending order by their fold-change values. Only statistically significant proteins (*p* < 0.05) with a fold-change ≥ 1.5 or ≤ −1.5 relative to the control were included. Albumin was excluded, due to high albumin concentration in the stimulation media. Color coded: ferroptosis, red; fatty acid metabolism, blueish; fatty acid degradation, green; PPAR signaling pathway, yellow

**Table 3:**
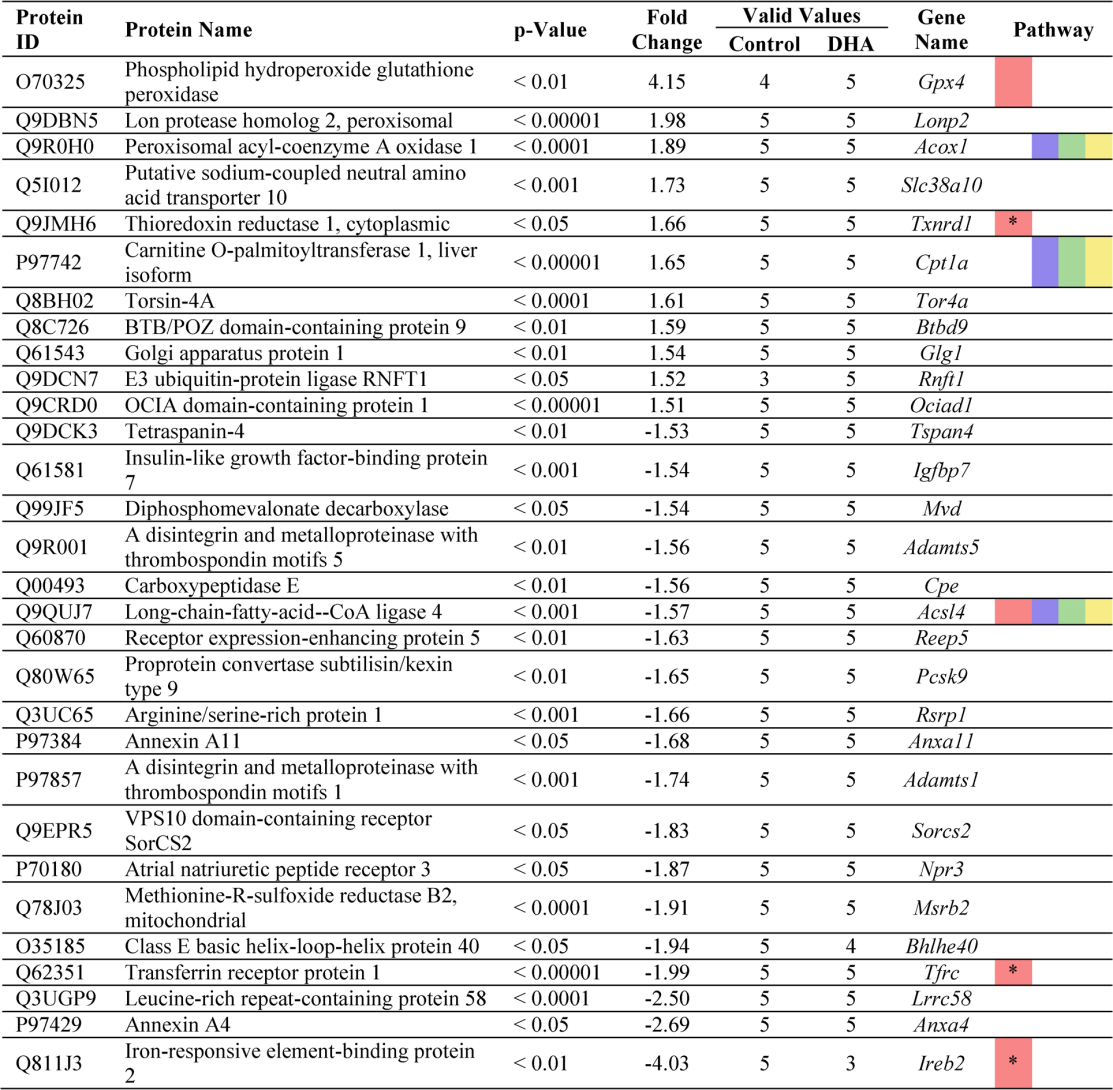
Differentially expression proteins in mesangial cells following DHA+AO stimulation, based on the analysis of the cellular proteome

This table lists the proteins, which are differentially expressed between the DHA + AO-stimulated cells and the control group, including their respective protein ID, name, p-value, fold-change, and gene name. The column “valid values” indicated the number of valid measurements for each protein, while only proteins with at least 3 valid measurements in both groups were included. The column “pathways” shows the relevant pathways associated with each protein, color-coded to match pathway categories presented in related analysis tables (Table 1). Proteins are sorted in descending order by their fold-change values. Only statistically significant proteins (*p* < 0.05) with a fold-change ≥ 1.5 or ≤ −1.5 relative to the control were included. Albumin was excluded, due to high albumin concentration in the stimulation media. Color coded: ferroptosis, red; fatty acid metabolism, blueish; fatty acid degradation, green; PPAR signaling pathway, yellow. *, proteins highly involved in the respective pathway, but are not identified through STRING analysis

The analysis of the signaling pathways revealed that ferroptosis is the most strongly regulated signaling pathway in all stimulation groups, with the highest signaling pathway strength (Table 1). Ferroptosis represents a distinct, regulated form of cell death that is driven by iron-dependent lipid peroxidation and differs from other forms of cell death such as necrosis or apoptosis. Important proteins involved in the regulation of ferroptosis include ACSL4, FTH1, TFRC, HMOX1 and GPX4. This metabolic pathway involves various mechanisms of iron accumulation that drive lipid peroxidation and increase intracellular reactive oxygen species (ROS), ultimately leading to membrane destruction and cell death. A detailed analysis of the ferroptosis mechanisms can be found in the discussion and is depicted in Fig. 6.

### Regulation of key ferroptosis-associated proteins indicates pro-ferroptotic effects of DHA and anti-ferroptotic effects of antioxidants

Interestingly, both DHA and oxDHA significantly upregulated proteins that drive ferroptosis (see Table 2 and Supp. Table 3). The expression of heme oxygenase 1 (HMOX1) was significantly increased in both the DHA-stimulated (fc: 1.98) and oxDHA-stimulated groups (fc: 1.87) compared to controls, but not in groups (co-)stimulated with antioxidants (see Table 3 and Supp. Tables 2 and 4). HMOX1 catalyzes the first, rate-limiting step of heme degradation, which increases the intracellular free iron content and consequently increases the overall rate of ferroptosis.

In contrast, the expression of glutathione peroxidase 4 (GPX4) was significantly increased in all antioxidant-treated groups, regardless of whether the antioxidants were administered alone (fc: 3.89) or in combination with DHA (fc: 4.15) or oxDHA (fc: 4.18). GPX4 is an essential component of the ferroptosis pathway and is considered one of the most protective proteins against ferroptosis and oxidative stress overall. It specifically catalyzes the reduction of phospholipid (hydro)peroxides to their non-toxic alcohol forms and thus prevents ferroptosis.

Similarly, Thioredoxin Reductase 1 (TXNRD1) was upregulated in all groups (co-)stimulated with antioxidants. Increased TXNRD1 expression was observed in the DHA + AO group (fc: 1.66), the oxDHA + AO group (fc: 1.67), and the AO-only group (fc: 1.9; Supp. Table 2). TXNRD1 plays a critical role in maintaining cellular redox balance by reducing oxidized thioredoxin, which in turn can reduce other proteins involved in mitigating oxidative stress.

Last, Iron-Responsive Element-Binding Protein 2 (IREB2) was significantly and relevantly downregulated in both the DHA + AO group (fc: −4.04) and the oxDHA + AO group (fc: −4.50), with a milder downregulation observed in the oxDHA-only group (fc: −1.96). IREB2 is a pivotal regulator in cellular iron metabolism, modulating the expression of iron-related proteins, including those responsible for iron storage and trafficking.

### Onset of self-protection mechanisms in ferroptosis

In addition to the protein regulations mentioned above, proteomic analysis showed that some additional proteins involved in ferroptosis were significantly and relevantly regulated by DHA and oxDHA, but not further significantly regulated by AO (see Tables 2 and Table 3 as well as Supp. Tables 3 and 4).

Firstly, transferrin receptor (TFRC) protein expression was decreased in the DHA-stimulated group (fc: −1.53), the oxDHA-stimulated group (fc: −2.00) and the two groups co-stimulated with antioxidants (DHA + AO and oxDHA + AO, with fc values of −1.99 and − 2.16, respectively). It is noteworthy that TFRC expression was also reduced in the carrier control group (fc: −1.95; Supp. Table 1). TFRC is an important regulator of cellular iron uptake.

Secondly, Ferritin Heavy Chain 1 (FTH1) showed upregulation in the DHA- and oxDHA-stimulated groups, as well as in the carrier-control group, with fc values of 1.61, ~ 1.50, and ~ 1.50, respectively. FTH1 is responsible for sequestering excess intracellular iron and thereby mitigating iron toxicity and oxidative stress.

Thirdly, Acyl-CoA Synthetase Long Chain Family Member 4 (ACSL4) was downregulated across four stimulation conditions. Decreased ACSL4 expression was observed in the DHA- and oxDHA-stimulated groups, as well as in the DHA + AO and oxDHA + AO co-stimulated groups, with corresponding fc values of −1.62, −1.53, −1.57, and − 1.53. ACSL4 plays a pivotal role in incorporating polyunsaturated fatty acids (PUFAs) into membrane phospholipids, which are particularly susceptible to peroxidation due to their polyunsaturated structure.

Together, these alterations hint at an onset of self-protection mechanisms in ferroptosis that is already underway.

### The expression of the ferroptosis-inhibiting protein glutathione peroxidase 4 was significantly increased by antioxidants, while ferritin induction is mediated by the carrier substance

**Fig. 3:**
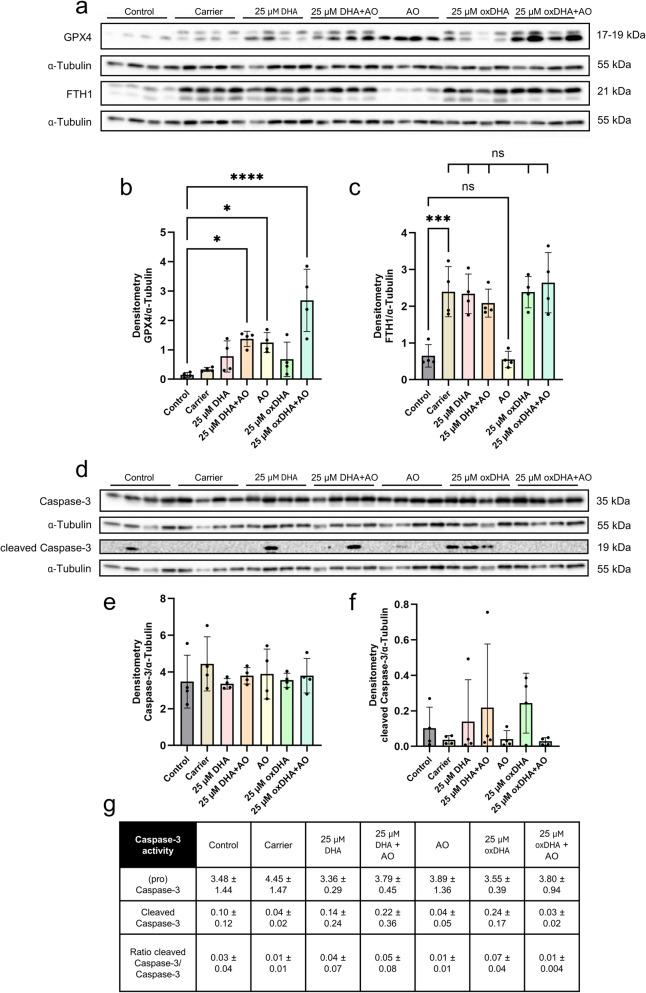
Western blot analysis of ferroptosis- and apoptosis-related proteins. **A** Representative Western blots showing GPX4, FTH1, and α-tubulin protein levels from cells treated with control medium, carrier control, 25 µM DHA, 25 µM DHA + AO (antioxidant), AO alone, 25 µM oxDHA, and 25 µM oxDHA + AO. α-Tubulin served as loading control. B + C: Densitometric analysis of (**B**) GPX4/α-tubulin and (**C**) FTH1/α-tubulin expression. In (**B**), all groups were compared to control; in (**C**), carrier-containing groups were additionally compared to the carrier control, to show carrier-related effects. Selective significant and non-significant comparisons are displayed. **D** Representative Western blots showing caspase-3, cleaved caspase-3, and α-tubulin from cells treated with control medium, carrier control, 25 µM DHA, 25 µM DHA + AO (antioxidant), AO alone, 25 µM oxDHA, and 25 µM oxDHA + AO. E + F: Densitometric quantification of (**E**) caspase-3 and (**F**) cleaved caspase-3, normalized to α-tubulin. All groups were compared to control. **G** Caspase-3 activity, expressed as the ratio of cleaved caspase-3 to total caspase-3. Data represent mean ± SD; *n* = 4 biological replicates. Statistical significance was determined by two-way ANOVA with Dunnett’s post-hoc test (*p < 0.05, **p < 0.01, ***p < 0.001, ****p < 0.0001)

Western Blot analysis of ferroptosis-related proteins revealed a significant increase in glutathione peroxidase 4 (GPX4) protein levels following antioxidant treatment, regardless of whether antioxidants were administered alone or in combination with DHA/oxDHA, Densiometric quantification normalized to α-tubulin demonstrated significantly elevated GPX4 expression in all antioxidant-treated groups compared to the control (Fig. 3a, b). These findings align with previously reported proteomic analyses of the cellular proteome, The consistent upregulation suggests that treatment with antioxidants enhances cellular defense against lipid peroxidation by inducing GPX4 expression.

Analysis of ferritin heavy chain 1 (FTH1) protein expression demonstrated a distinct regulation pattern. FTH1 protein levels were significantly elevated in all groups containing the carrier solution, with no statistical differences observed between the various carrier-treated groups (Fig. 3a, c). This indicates that FTH1 induction is primarily mediated by the carrier vehicle rather than the experimental treatments.

### The ratio of cleaved caspase 3 to total caspase 3 excludes a relevant contribution of apoptosis to DHA-induced cell death

Western blot analysis revealed no significant changes in the expression of total caspase-3 or cleaved caspase-3 in all treatment groups after normalization to α-tubulin (Fig. 3d, e, f). To further evaluate potential apoptotic activation, the caspase-3 activity ratio was calculated as cleaved caspase-3 to total (pro) caspase-3 (Fig. 3g). This analysis demonstrated no statistically significant differences between experimental conditions. The maximum observed mean caspase-3 activation ratio was 0.07, indicating minimal proteolytic activation. According to established criteria in comparable studies [19] ratios of approximately 0.8 to 1.0 are generally considered indicative of apoptosis. Nonetheless, the consistent detection of cleaved caspase-3 in multiple oxDHA-treated samples suggests potential low-level apoptotic activity, although the lack of statistical significance precludes definitive conclusions. A contribution of apoptosis to oxDHA-induced cytotoxicity, while likely minor, cannot be entirely excluded.

### Ferrostatin prevents DHA-induced loss of mesangial cell viability

Since our analyses suggested a possible link between ferroptosis-related network activations and increased cell death following DHA stimulation, we next investigated whether ferroptosis is indeed the mechanism underlying DHA-induced cell death using a multi-step approach. Therefore, we first tested the effect of ferrostatin-1 (in the following ferrostatin), a known ferroptosis inhibitor, in the following experimental setup: metabolic activity was measured *via* MTT in (1) mesangial cells (control group), (2) DHA-stimulated mesangial cells, (3) DHA + AO- stimulated mesangial cells, (4) DHA + ferrostatin- stimulated mesangial cells, (5) ferrostatin-stimulated mesangial cells, and (6) negative control (no cells).

**Fig. 4:**
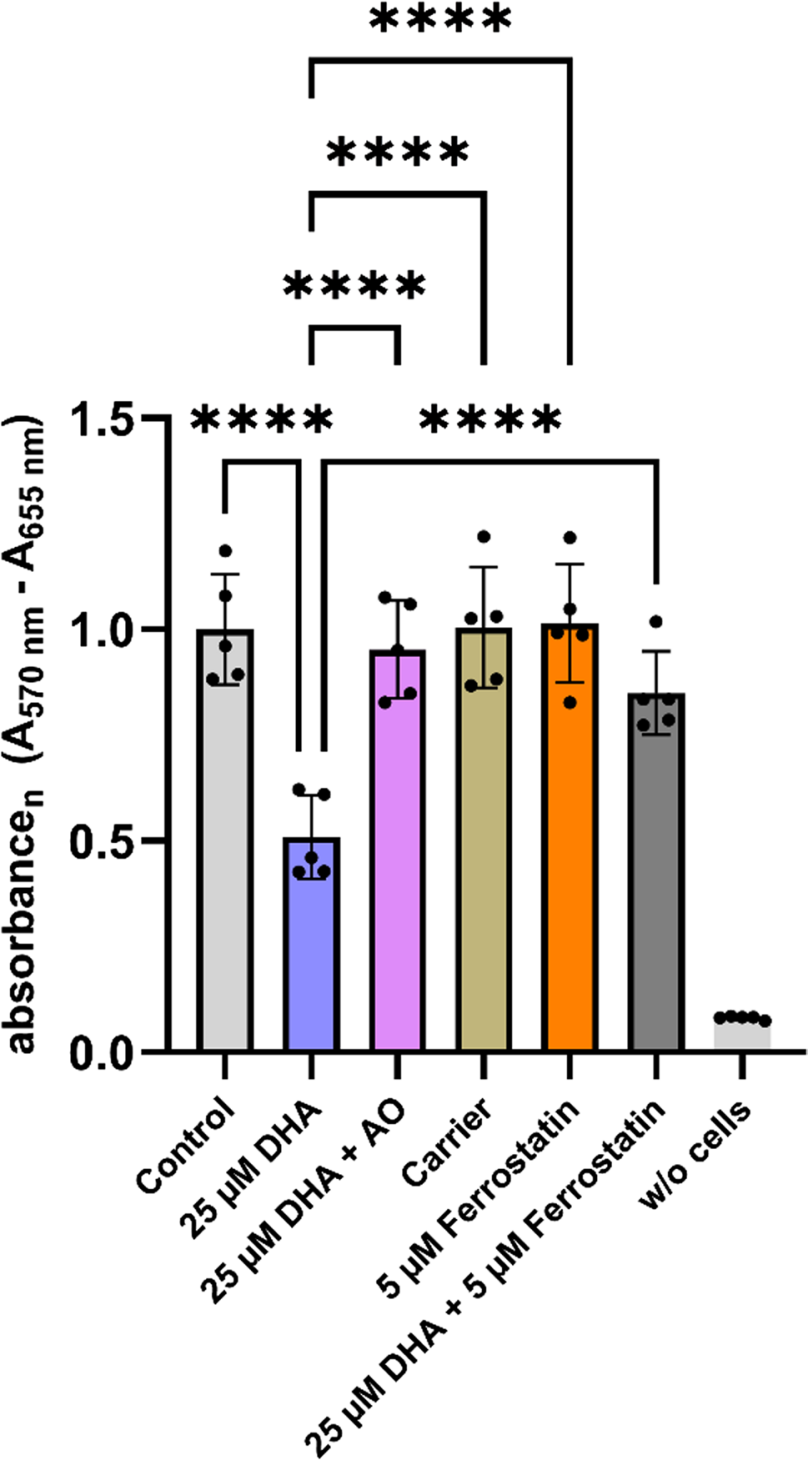
Impact of ferrostatin treatment on metabolic activity. MTT assay results assessing metabolic activity under different treatment conditions. Depicted as relative absorbance values measured at A570nm – A655nm normalized to the control group. Bars represent mean ± SD (*n* = 5, biological replicates). Statistical analysis was performed using two-way ANOVA and post-hoc Tukey’s test for multiple comparisons was conducted. Significant differences are indicated by asterisks (*, *p* < 0.05; **, *p* < 0.01; ***, *p* < 0.001; ****, *p* < 0.0001). Not all pairwise comparisons are shown. For each biological replicate, six technical replicates were measured and averaged

Cells stimulated with 25 µM DHA alone exhibited a marked decrease in metabolic activity compared to the control group (DHA: 0.51 ± 0.10 vs. Control: 1.00 ± 0.13; *p* < 0.0001; mean ± SD; *n* = 5 for all groups), consistent with increased cell death observed in our prior studies. The addition of an antioxidant cocktail (DHA + AO) almost completely rescued this effect, significantly improving metabolic activity relative to DHA alone (DHA + AO: 0.95 ± 0.12 vs. DHA: 0.51 ± 0.10; *p* < 0.0001), reaching levels statistically similar to the control (*p* > 0.05). Cells treated with ferrostatin alone (5 µM) displayed metabolic activity levels comparable to the control (ferrostatin: 1.01 ± 0.17; vs. Control: 1.00 ± 0.13; *p* > 0.05), indicating that ferrostatin itself does not affect cell viability. Importantly, co-treatment with DHA and ferrostatin (DHA + 5 µM ferrostatin) significantly preserved metabolic activity compared to DHA alone (DHA + ferrostatin: 0.85 ± 0.10 vs. DHA: 0.51 ± 0.10; *p* < 0.0001), approaching control levels (DHA + ferrostatin vs. Control, *p* > 0.05). These data initially support the hypothesis that DHA-induced cell death could be mediated by ferroptosis (Fig. 4).

### In line with its ferroptotic-inducing effect DHA induces strong lipid peroxidation

**Fig. 5:**
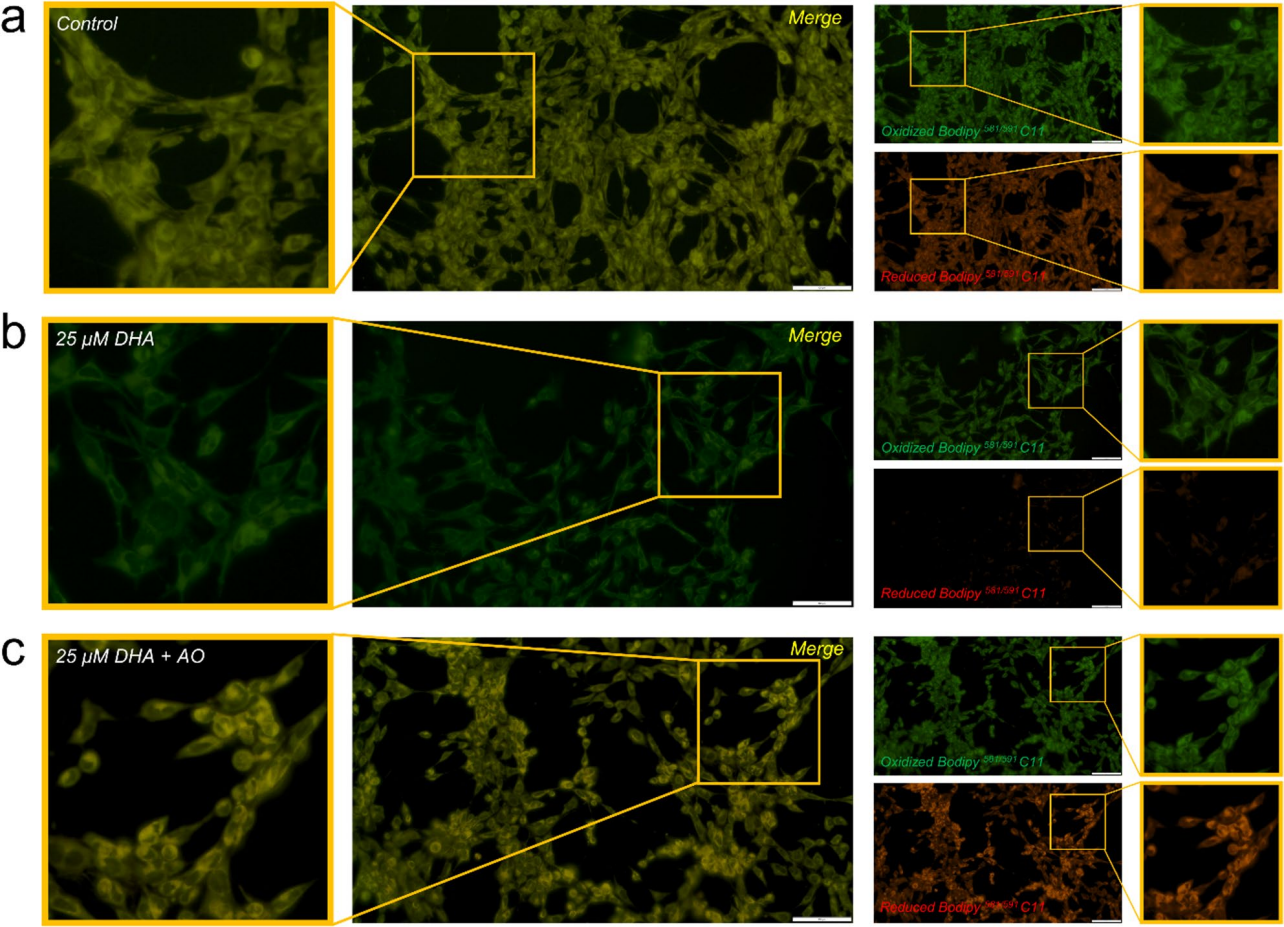
DHA-induced lipid peroxidation detected by Bodipy^581/591^ C‑11 staining. Representative fluorescence microscopy images of cells stained with the lipid peroxidation sensor Bodipy^581/591^ C‑11 under the following conditions: control (**A**), 25 µM DHA (**B**), and 25 µM DHA + AO (**C**). For each condition, the merged overlay (left) and individual oxidized (green, right, top) and reduced (red, right, bottom) channels are shown. For better visualization of the individual channels, brightness was uniformly increased (oxidized signal: +15%; reduced signal: +30%) without contrast adjustment. Merged images represent unmodified overlays. Scale bar: 100 μm

To further confirm that ferroptosis causes DHA-induced cell death *via* lipid peroxidation, we performed immunofluorescence staining. Lipid peroxidation was assessed in living cells using Bodipy^581/591^ C-11 staining. In the control group, the cells showed both red (reduced) and green (oxidized) signals, resulting in a yellow, merged image indicating basal peroxidation levels (Fig. 5a). Treatment with DHA resulted in a visible increase in green fluorescence with a simultaneous reduction in the red signal, producing a predominantly green, merged image consistent with increased lipid peroxidation (Fig. 5b). This effect was significantly attenuated by simultaneous treatment with antioxidants (DHA + AO), restoring the strong red fluorescence and producing a yellow, slightly orange composite image (Fig. 5c). The absence of relevant apoptosis activity (caspase-3 blot), the induction of GPX4 by antioxidants, and the restoration of cell viability by ferrostatin, together with evidence of lipid peroxidation (Bodipy^581/591^ C-11 staining), support the conclusion that DHA-induced cell death of mesangial cells is due to ferroptosis.

## Discussion

Our study focused on investigating the molecular effects of DHA treatment on cultured mesangial cells. We found that DHA alone impairs cell viability, but the synergistic interaction of DHA and antioxidants represents a promising approach for therapeutic modulation of mesangial cell function. In particular, our results show that (i) DHA induces mesangial cell death through an oxidant-mediated mechanism, (ii) DHA causes an imbalance in the expression of pro- and anti-ferroptotic effector molecules, (iii) the ferroptosis-regulating protein GPX4 is induced by antioxidants, (iv) the ferroptosis-specific inhibitor ferrostatin-1 restores the viability of mesangial cells under DHA treatment, and finally (v) DHA treatment is associated with lipid peroxidation in mesangial cells. The absence of significant caspase-3 activation argues against apoptosis as a relevant cell death mechanism. The mechanistic link between DHA and ferroptosis in mesangial cells could have important implications for future therapeutic strategies aimed at maintaining and promoting kidney health while preventing kidney diseases in which mesangial cell viability is compromised.

In detail, contrary to expectations and previous studies, stimulation of mesangial cells with DHA alone led to a significant reduction in viability and ultimately cell death. Concomitant treatment with DHA and antioxidants almost completely prevented DHA-induced cell death, indicating a protective effect of antioxidants. To further explore the underlying molecular mechanisms, we performed proteomic profiling which revealed altered expression of proteins associated with ferroptosis. Further, combined treatment of cells with DHA and antioxidants resulted in a distinct proteomic signature with a remarkable upregulation of proteins involved in cell metabolism and anti-inflammatory responses.

Ferroptosis is a regulated form of cell death driven by iron-dependent oxidative stress and phospholipid peroxidation, setting it apart from other types of programmed cell death like apoptosis [20]. Mesangial cells are crucial for structural support and cellular communication within the glomerulus, but they are highly susceptible to ferroptosis. Therefore, an imbalance in the expression of proteins important for regulating ferroptosis can lead to self-perpetuating cell damage once it has begun. Hence, this high vulnerability of mesangial cells to ferroptosis has critical implications for glomerular diseases [12, 21].

**Fig. 6:**
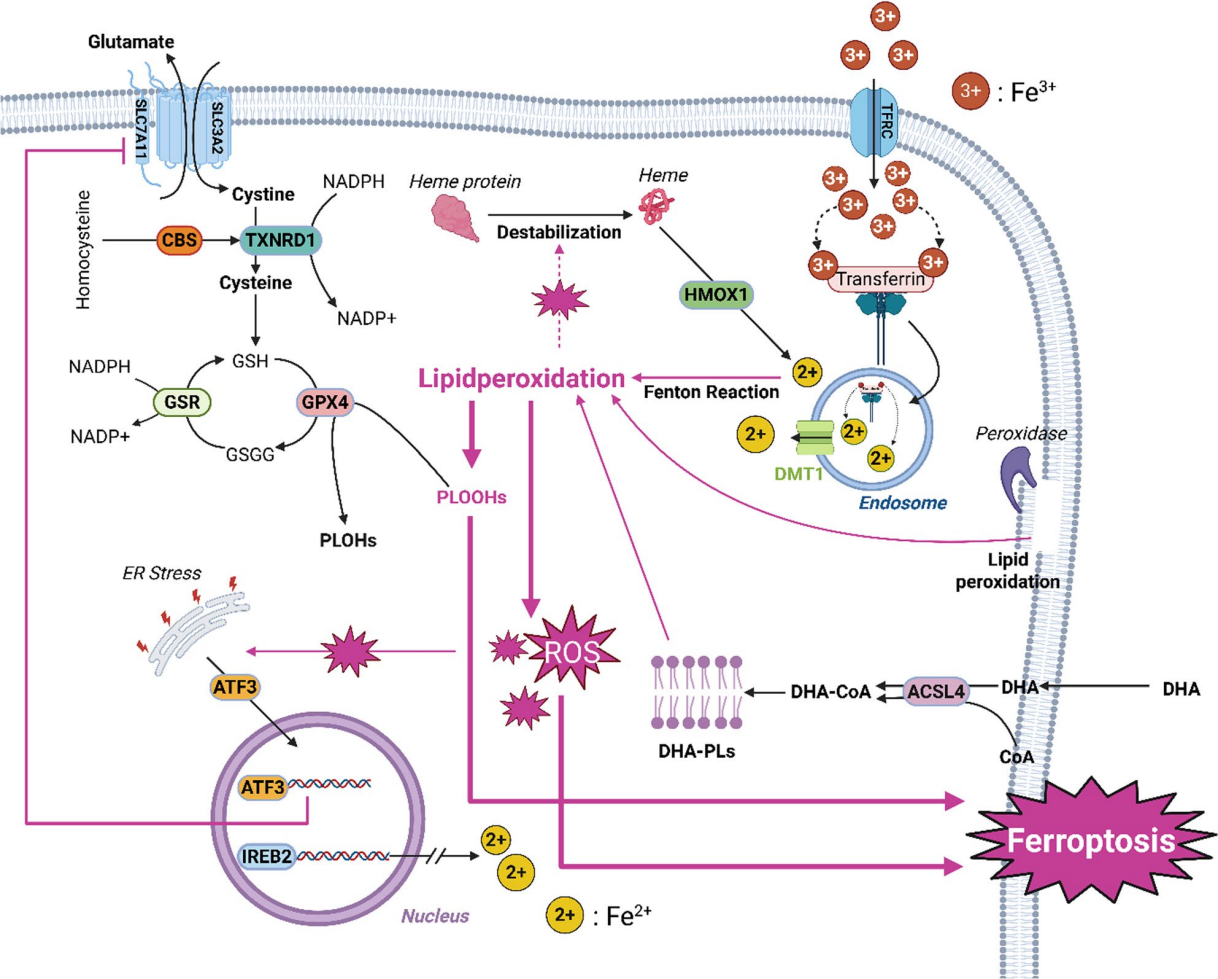
Mechanisms of ferroptosis and contributing factors inside of the cell. The figure shows central mechanism of ferroptosis, a form of programmed cell death triggered by iron-dependent lipid peroxidation. The import of trivalent iron (Fe3+) through the transferrin receptor (TFRC) and its reduction to its bivalent (Fe2+) form leads to iron accumulation in the endosome and its release into the cytosol via DMT1. Free iron catalyzes the formation of ROS, which drive lipid peroxidation. This results in peroxidized lipid derivates (PLOOHHs), which lead to cell damage and ferroptosis. Key molecules such as GPX4 and TXNRD1 show antioxidative and therefore anti-ferroptotic effects while ACSL4 increases susceptibility to ferroptosis. Transcription factors such as ATF3 and IREB2 regulate the expression of ferroptosis-associated genes. Figure created with BioRender.com

Key proteins involved in the regulation of ferroptosis include TFRC [22], HMOX1 [23, 24], ACSL4 [25], TXNRD1, GPX4 [26] and IREB2, all of which contribute to a tightly coordinated network that influences iron homeostasis, lipid peroxidation, and redox balance. Together they represent checkpoint molecules that can determine whether cells undergo ferroptosis or maintain their cellular integrity (Fig. 6) [27]. While TFRC, HMOX1, and ACSL4 promote ferroptosis, TXNRD1 (thioredoxin reductase 1) and GPX4 (glutathione peroxidase 4) serve as important anti-ferroptotic proteins that balance this process by maintaining redox homeostasis. Interestingly, HMOX1 is the only pro-ferroptotic protein that is upregulated by DHA, while the ability of ferrostatin-1 to effectively attenuate cell death after DHA treatment clearly shows that cells die by ferroptosis under DHA. The downregulation of TFRC and ACSL4 could indicate the onset of counterregulatory mechanisms in the cell for self-protection, but there is a complete lack of upregulation of anti-ferroptotic proteins.

Specifically, ferroptosis begins with iron uptake, which is mediated by the transferrin receptor (TFRC) on the cell membrane. TFRC facilitates the influx of iron into the cell and increases the intracellular Fe³⁺ (ferric iron) level. In the cell, Fe³⁺ is subsequently reduced to Fe²⁺ (ferrous iron), a highly reactive form that drives the Fenton reaction and generates reactive oxygen species. The downregulation of TFRC thus has a braking effect on ROS production. ROS, in turn, trigger lipid peroxidation, leading to the formation of lipid hydroperoxides, which play a central role in the execution of ferroptosis [28–30].When ROS accumulate, they destabilize cellular heme proteins and release free heme, which is then degraded by HMOX1 (heme oxygenase 1), a protein upregulated by DHA. This degradation process generates additional Fe²⁺, which further increases ROS production. The upregulation of HMOX1 therefore creates a positive feedback loop that accelerates lipid peroxidation and ferroptotic cell death [21]. This self-amplifying cycle is a hallmark of ferroptosis and underscores the potential for ferroptosis to be a self-propagating form of cell death once initiated.

In parallel to ferroptosis processes, ACSL4 (acyl-CoA synthetase long-chain family member 4) plays a crucial role by incorporating polyunsaturated fatty acids, including docosahexaenoic acid, into phospholipids, forming DHA-phospholipids (DHA-PLs). These DHA-PLs are particularly vulnerable to peroxidation, making them prime targets for ROS. The peroxidation of these phospholipids is a critical step that further drives the ferroptotic pathway, underscoring ACSL4’s role as a pro-ferroptotic factor. Downregulation of ACSL4 by DHA may protect the cell from excessive n-3 PUFA uptake and finally from ferroptosis.

Co-treatment of cells with antioxidants rescues mesangial cells from DHA-induced ferroptosis. At the molecular level, antioxidants upregulate the expression of three proteins (TXNRD1, GPX4, IREB2) that are very likely involved in this protective effect. TXNRD1 acts as an antioxidant by helping to maintain the cell’s redox balance, while GPX4 is considered the primary ferroptosis inhibitor, as it directly reduces lipid hydroperoxides, preventing their accumulation and thereby interrupting the ferroptotic cascade. IREB2 (iron-responsive element-binding protein 2) modulates ferroptosis indirectly by controlling the expression of iron metabolism genes, thereby regulating cellular iron levels and sensitivity to ferroptosis. By adjusting intracellular iron concentrations, IREB2 helps fine-tune the balance between iron availability and the risk of initiating ferroptotic cell death.

While our findings provide mechanistic insights into DHA-induced ferroptosis in mesangial cells, several limitations must be acknowledged. First, the use of murine (rather than human) mesangial cells may limit translational relevance, as species-specific differences in lipid metabolism and antioxidant responses exist. Second, the simplified in vitro system lacks the metabolic interaction of intact kidney tissue and does not fully replicate the renal microenvironment. Although serum-free conditions would allow for more precise control of free DHA concentrations, we deliberately maintained serum-containing conditions to better mimic the physiological situation in which fatty acids are bound to proteins. The potential variability introduced by serum components was minimized by using a single batch for all media preparations. Finally, the use of a broad-spectrum antioxidant cocktail instead of a lipid-specific antioxidant limits mechanistic precision. While this approach effectively models a general therapeutic antioxidant strategy, it does not allow us to distinguish protection through inhibition of lipid peroxidation from other antioxidant mechanisms. The use of more specific inhibitors in future studies would help refine our understanding of the exact pathways involved.

Putting our findings into a clinical context, there is increasing evidence that ferroptosis is a key factor in renal fibrosis and the progression of chronic kidney disease (CKD) [31]. Although n-3 PUFAs, such as DHA, show well-documented anti-inflammatory effects in clinical studies, their pro-ferroptotic activity, as shown here in mesangial cells, reveals a paradoxical duality in their therapeutic potential. This dichotomy may explain why study results to date do not permit a general recommendation for n-3 PUFA supplementation in CKD, as the net benefit likely depends on the redox buffering capacity of the individual. Indeed, variability in oxidative stress responses between patients may account for the inconsistent results observed in trials of n-3 PUFAs [32, 33].

Oxidative stress in particular is a key modulator of cellular vulnerability, not only in CKD but also during early development, especially in the kidneys. Preterm infants, for example, have reduced antioxidative capacities, and perinatal interventions such as oxygen supplementation or nutritional support can further exacerbate oxidative stress [34]. The role of oxidative stress in both CKD and developing kidneys underscores the broad relevance of ferroptosis across life stages, suggesting shared mechanistic vulnerabilities. Targeting ferroptosis, e.g. by antioxidant treatments or ferrostatin-1, could act synergistically with n-3 PUFA therapies to preserve renal function by (i) attenuating DHA-induced lipid peroxidation while preserving anti-inflammatory benefits, (ii) interrupting the ferroptosis-fibrosis axis, or (iii) enabling redox-based patient stratification to identify patients who respond to therapy [27, 31, 35].

Although our mechanistic findings suggest promising approaches for personalized n-3 PUFA therapy, further studies, especially in primary cells or in vivo models, are needed to address remaining translational challenges, including the optimal timing for intervention and patient-specific redox thresholds.

## Conclusion

In summary, the available data show that DHA alone causes mesangial cell death *via* a ferroptosis-dependent mechanism. However, simultaneous treatment with antioxidants prevents DHA-induced mesangial cell death. Overall, these data not only provide new mechanistic insights into the regulatory networks in mesangial cells during treatment with DHA, but also relevant aspects that should be considered when planning and evaluating future clinical trials testing the effects of n-3 PUFAs. 

## Supplementary Information

Supplementary Material 1.

Supplementary Material 2.

## Data Availability

The mass spectrometry proteomics data have been deposited to the ProteomeXchange Consortium via the PRIDE partner repository with the dataset identifier PXD065885. Log in to the PRIDE website using the following details: Project accession: PXD065885; Token: fmbAdb5ccrFc.

## Abbreviations

ACSL4: Acyl-CoA Synthetase Long Chain Family Member 4
AO: Antioxidants, Antioxidant-Cocktail
BSA: Bovine serum albumin
CAA: Chloroacetamide
CECAD: Cologne Excellence Cluster for Aging and Aging-Associated Diseases
CKD: Chronic kidney disease
DHA: Docosahexaenoic acid
DIA-NN: Data-independent acquisition mode
DTT: Dithiothreitol
Fc: Fold-change
FTH1: Ferritin heavy chain
GPX4: Glutathione peroxidase
HMOX1: Heme oxygenase 1
IREB2: Iron-responsive element-binding protein 2
IUGR: Intrauterine growth restriction
LC-MS/MS: Liquid Chromatography-Tandem Mass Spectrometry
LysC: Lysyl endopeptidase
MNR: Milan Normotensive Rats
n-3 PUFA: Omega-3 polyunsaturated fatty acids
oxDHA: oxidized docosahexaenoic acid
PL: Phospholipid
ROS: Reactive oxygen species
TEAB: Tetraethylammonium bromide
TFRC: Transferrin receptor
TXNRD1: Thioredoxin reductase 1
