# Supplementary material for: Antioxidants rescue murine mesangial cells from docosahexaenoic acid-induced ferroptosis
Source: Mol Cell Pediatr. 2026 Jan 5;13:2. doi: 10.1186/s40348-025-00215-y (PMC12770095; doi:10.1186/s40348-025-00215-y)
Supplement: Supplementary file 1 — Supplementary Material 1. [file 40348_2025_215_MOESM1_ESM.docx]

| **Protein ID** | **Protein Name** | **p-Value** | **Fold Change** | **Valid Values** | | **Gene Name** | **Pathway** | | | |
| --- | --- | --- | --- | --- | --- | --- | --- | --- | --- | --- |
|  |  |  |  | **Control** | **Carrier** |  |  |  |  |  |
| Q62351 | Transferrin receptor protein 1 | < 0.00001 | -1.95 | 5 | 5 | *Tfrc* |  |  |  |  |
| Q9EPR5 | VPS10 domain-containing receptor SorCS2 | < 0.001 | -2.00 | 5 | 5 | *Sorcs2* |  |  |  |  |
| Q811J3 | Iron-responsive element-binding protein 2 | < 0.01 | -4.38 | 5 | 3 | *Ireb2* | * |  |  |  |
| P17809 | Solute carrier family 2. facilitated glucose transporter member 1 | < 0.05 | -1.78 | 5 | 5 | *Slc2a1* |  |  |  |  |
| Q3U1F9 | Phosphoprotein associated with glycosphingolipid-enriched microdomains 1 | < 0.05 | -3.06 | 5 | 3 | *Pag1* |  |  |  |  |
| P23336 | N-acetyllactosaminide alpha-1.3-galactosyltransferase | < 0.05 | -1.98 | 5 | 3 | *Ggta1* |  |  |  |  |

**Supplementary Table 1: Differentially expression proteins in mesangial cells following stimulation with the carrier, based on the analysis of the cellular proteome.**

*This table lists the proteins, which are differentially expressed between the carrier-stimulated cells and the control group, including their respective protein ID, name, p-value, fold-change, and gene name. The column “valid values” indicated the number of valid measurements for each protein, while only proteins with at least 3 valid measurements in both groups were included. The column “pathways” shows the relevant pathways associated with each protein, color-coded to match pathway categories presented in related analysis tables (Table 1). Proteins are sorted in descending order by their fold-change values. Only statistically significant proteins (p < 0.05) with a fold-change ≥ 1.5 or ≤ -1.5 relative to the control were included. Albumin was excluded, due to high albumin concentration in the stimulation media. Color coded: ferroptosis, red; fatty acid metabolism, blueish; fatty acid degradation, green; PPAR signaling pathway, yellow. *, proteins highly involved in the respective pathway, but are not identified through STRING analysis.*

| **Protein ID** | **Protein Name** | **p-Value** | **Fold Change** | **Valid Values** | | **Gene Name** | **Pathway** | | | |
| --- | --- | --- | --- | --- | --- | --- | --- | --- | --- | --- |
|  |  |  |  | **Control** | **AO** |  |  |  |  |  |
| Q3UGP9 | Leucine-rich repeat-containing protein 58 | < 0,00001 | -2,66 | 5 | 5 | *Lrrc58* |  |  |  |  |
| P18406 | CCN family member 1 | < 0,01 | -1,51 | 5 | 5 | *Ccn1* |  |  |  |  |
| O70325 | Phospholipid hydroperoxide glutathione peroxidase | < 0,01 | 3,89 | 4 | 5 | *Gpx4* |  |  |  |  |
| Q9QZA0 | Carbonic anhydrase 5B, mitochondrial | < 0,01 | 1,72 | 5 | 5 | *Ca5b* |  |  |  |  |
| Q9JMH6 | Thioredoxin reductase 1, cytoplasmic | < 0,05 | 1,90 | 5 | 5 | *Txnrd1* | * |  |  |  |

***Supplementary Table 2: Differentially expression proteins in mesangial cells following antioxidant stimulation, based on the analysis of the cellular proteome***.

*This table lists the proteins, which are differentially expressed between the AO-stimulated cells and the control group, including their respective protein ID, name, p-value, fold-change, and gene name. The column “valid values” indicated the number of valid measurements for each protein, while only proteins with at least 3 valid measurements in both groups were included. The column “pathways” shows the relevant pathways associated with each protein, color-coded to match pathway categories presented in related analysis tables (Table 1). Proteins are sorted in descending order by their fold-change values. Only statistically significant proteins (p < 0.05) with a fold-change ≥ 1.5 or ≤ -1.5 relative to the control were included. Albumin was excluded, due to high albumin concentration in the stimulation media. Color coded: ferroptosis, red; fatty acid metabolism, blueish; fatty acid degradation, green; PPAR signaling pathway, yellow. *, proteins highly involved in the respective pathway, but are not identified through STRING analysis.*

| **Protein ID** | **Protein Name** | **p-Value** | **Fold Change** | **Valid Values** | | **Gene Name** | **Pathway** | | | |
| --- | --- | --- | --- | --- | --- | --- | --- | --- | --- | --- |
|  |  |  |  | **Control** | **oxDHA** |  |  |  |  |  |
| P43883 | Perilipin-2 | < 0.05 | 2.97 | 4 | 5 | *Plin2* |  |  |  |  |
| Q9DBN5 | Lon protease homolog 2, peroxisomal | < 0.00001 | 2.08 | 5 | 5 | *Lonp2* |  | | | |
| Q9R0H0 | Peroxisomal acyl-coenzyme A oxidase 1 | < 0.00001 | 2.08 | 5 | 5 | *Acox1* |  |  |  |  |
| P14901 | Heme oxygenase 1 | < 0.05 | 1.87 | 5 | 5 | *Hmox1* |  |  |  |  |
| Q61543 | Golgi apparatus protein 1 | < 0.001 | 1.81 | 5 | 5 | *Glg1* |  | | | |
| Q9JJV2 | Profilin-2 | < 0.01 | 1.81 | 5 | 5 | *Pfn2* |  | | | |
| P62827 | GTP-binding nuclear protein Ran | < 0.05 | 1.80 | 5 | 5 | *Ran* |  | | | |
| O88783 | Coagulation factor V | < 0.05 | 1.74 | 5 | 5 | *F5* |  | | | |
| P14069 | Protein S100-A6 | < 0.05 | 1.70 | 5 | 5 | *S100a6* |  | | | |
| Q8BHL8 | Proteasome inhibitor PI31 subunit | < 0.05 | 1.68 | 5 | 5 | *Psmf1* |  | | | |
| Q8BXA1 | Golgi integral membrane protein 4 | < 0.001 | 1.64 | 5 | 5 | *Golim4* |  | | | |
| P45377 | Aldose reductase-related protein 2 | < 0.05 | 1.61 | 5 | 5 | *Akr1b8* |  | | | |
| P48758 | Carbonyl reductase [NADPH] 1 | < 0.05 | 1.60 | 5 | 5 | *Cbr1* |  | | | |
| Q9CQI3 | Glia maturation factor beta | < 0.05 | 1.58 | 5 | 5 | *Gmfb* |  | | | |
| Q62159 | Rho-related GTP-binding protein RhoC | < 0.05 | 1.52 | 5 | 5 | *Rhoc* |  | | | |
| P50543 | Protein S100-A11 | < 0.05 | 1.52 | 5 | 5 | *S100a11* |  | | | |
| P35441 | Thrombospondin-1 | < 0.01 | -1.51 | 5 | 5 | *Thbs1* |  | | | |
| Q91X97 | Neurocalcin-delta | < 0.01 | -1.51 | 5 | 5 | *Ncald* |  | | | |
| Q9R1S3 | GPI ethanolamine phosphate transferase 1 | < 0.05 | -1.52 | 5 | 4 | *Pign* |  | | | |
| Q9QUJ7 | Long-chain-fatty-acid--CoA ligase 4 | < 0.001 | -1.53 | 5 | 5 | *Acsl4* |  |  |  |  |
| Q64310 | Surfeit locus protein 4 | < 0.05 | -1.53 | 5 | 5 | *Surf4* |  | | | |
| Q9QZI8 | Serine incorporator 1 | < 0.01 | -1.54 | 5 | 5 | *Serinc1* |  | | | |
| Q8BVF7 | Gamma-secretase subunit APH-1A | < 0.01 | -1.56 | 5 | 5 | *Aph1a* |  | | | |
| Q8R2E9 | ERO1-like protein beta | < 0.05 | -1.56 | 5 | 5 | *Ero1b* |  | | | |
| Q9EPT5 | Solute carrier organic anion transporter family member 2A1 | < 0.001 | -1.59 | 5 | 5 | *Slco2a1* |  | | | |
| Q9ERY9 | Ergosterol biosynthetic protein 28 homolog | < 0.05 | -1.60 | 5 | 5 | *Erg28* |  | | | |
| Q78J03 | Methionine-R-sulfoxide reductase B2, mitochondrial | < 0.01 | -1.62 | 5 | 5 | *Msrb2* |  | | | |
| Q8VDP6 | CDP-diacylglycerol--inositol 3-phosphatidyltransferase | < 0.05 | -1.62 | 5 | 5 | *Cdipt* |  | | | |
| Q9DCK3 | Tetraspanin-4 | < 0.01 | -1.65 | 5 | 5 | *Tspan4* |  | | | |
| Q9JI57 | General transcription factor II-I repeat domain-containing protein 1 | < 0.01 | -1.68 | 5 | 5 | *Gtf2ird1* |  | | | |
| Q62351 | Transferrin receptor protein 1 | < 0.0001 | -1.69 | 5 | 5 | *Tfrc* |  |  |  |  |
| P27889 | Hepatocyte nuclear factor 1-beta | < 0.01 | -1.71 | 5 | 5 | *Hnf1b* |  | | | |
| Q08AT1 | Ras-like protein family member 12 | < 0.05 | -1.76 | 5 | 5 | *Rasl12* |  | | | |
| Q3UC65 | Arginine/serine-rich protein 1 | < 0.01 | -1.77 | 5 | 5 | *Rsrp1* |  | | | |
| Q811J3 | Iron-responsive element-binding protein 2 | < 0.01 | -1.96 | 5 | 5 | *Ireb2* |  | | | |
| Q60870 | Receptor expression-enhancing protein 5 | < 0.01 | -2.22 | 5 | 5 | *Reep5* |  | | | |
| Q9CZY2 | Transcription elongation factor A protein-like 8 | < 0.05 | -2.26 | 5 | 5 | *Tceal8* |  | | | |
| P13516 | Acyl-CoA desaturase 1 | < 0.01 | -2.70 | 5 | 5 | *Scd1* |  |  |  |  |
| Q3UHH2 | Solute carrier family 22 member 23 | < 0.05 | -3.10 | 5 | 3 | *Slc22a23* |  | | | |

***Supplementary Table 3: Differentially expression proteins in mesangial cells following oxDHA stimulation, based on the analysis of the cellular proteome.***

*This table lists the proteins, which are differentially expressed between the oxDHA-stimulated cells and the control group, including their respective protein ID, name, p-value, fold-change, and gene name. The column “valid values” indicated the number of valid measurements for each protein, while only proteins with at least 3 valid measurements in both groups were included. The column “pathways” shows the relevant pathways associated with each protein, color-coded to match pathway categories presented in related analysis tables (Table 1). Proteins are sorted in descending order by their fold-change values. Only statistically significant proteins (p < 0.05) with a fold-change ≥ 1.5 or ≤ -1.5 relative to the control were included. Albumin was excluded, due to high albumin concentration in the stimulation media. Color coded: ferroptosis, red; fatty acid metabolism, blueish; fatty acid degradation, green; PPAR signaling pathway, yellow. *, proteins highly involved in the respective pathway, but are not identified through STRING analysis.*

| **Protein ID** | **Protein Name** | **p-Value** | **Fold Change** | **Valid Values** | | **Gene Name** | **Pathway** | | | |
| --- | --- | --- | --- | --- | --- | --- | --- | --- | --- | --- |
|  |  |  |  | **Control** | **oxDHA** |  |  |  |  |  |
| O70325 | Phospholipid hydroperoxide glutathione peroxidase | < 0.01 | 4.18 | 4 | 5 | *Gpx4* |  |  |  |  |
| Q9DBN5 | Lon protease homolog 2, peroxisomal | < 0.0001 | 1.83 | 5 | 5 | *Lonp2* |  | | | |
| Q9R0H0 | Peroxisomal acyl-coenzyme A oxidase 1 | < 0.0001 | 1.72 | 5 | 5 | *Acox1* |  |  |  |  |
| Q5I012 | Putative sodium-coupled neutral amino acid transporter 10 | < 0.001 | 1.71 | 5 | 5 | *Slc38a10* |  | | | |
| Q9JMH6 | Thioredoxin reductase 1, cytoplasmic | < 0.05 | 1.67 | 5 | 5 | *Txnrd1* |  | | | |
| P97742 | Carnitine O-palmitoyltransferase 1, liver isoform | < 0.00001 | 1.63 | 5 | 5 | *Cpt1a* |  |  |  |  |
| Q8BXA1 | Golgi integral membrane protein 4 | < 0.001 | 1.63 | 5 | 5 | *Golim4* |  | | | |
| Q61543 | Golgi apparatus protein 1 | < 0.01 | 1.62 | 5 | 5 | *Glg1* |  | | | |
| Q91WE6 | Threonylcarbamoyladenosine tRNA methylthiotransferase | < 0.05 | 1.58 | 5 | 5 | *Cdkal1* |  | | | |
| Q8C726 | BTB/POZ domain-containing protein 9 | < 0.01 | 1.52 | 5 | 5 | *Btbd9* |  | | | |
| Q9EQG3 | Sciellin | < 0.05 | 1.51 | 5 | 5 | *Scel* |  | | | |
| Q8BH02 | Torsin-4A | < 0.01 | 1.50 | 5 | 5 | *Tor4a* |  | | | |
| O08528 | Hexokinase-2 | < 0.00001 | -1.52 | 5 | 5 | *Hk2* |  | | | |
| P16675 | Lysosomal protective protein | < 0.05 | -1.52 | 5 | 5 | *Ctsa* |  | | | |
| Q9QUJ7 | Long-chain-fatty-acid--CoA ligase 4 | < 0.0001 | -1.53 | 5 | 5 | *Acsl4* |  |  |  |  |
| Q9JKZ2 | Sodium/myo-inositol cotransporter | < 0.05 | -1.53 | 5 | 5 | *Slc5a3* |  | | | |
| Q9DBY1 | E3 ubiquitin-protein ligase synoviolin | < 0.05 | -1.55 | 5 | 5 | *Syvn1* |  | | | |
| Q9QZI8 | Serine incorporator 1 | < 0.01 | -1.58 | 5 | 5 | *Serinc1* |  | | | |
| Q9DCK3 | Tetraspanin-4 | < 0.01 | -1.59 | 5 | 5 | *Tspan4* |  | | | |
| Q9EPT5 | Solute carrier organic anion transporter family member 2A1 | < 0.01 | -1.61 | 5 | 5 | *Slco2a1* |  | | | |
| Q00493 | Carboxypeptidase E | < 0.01 | -1.61 | 5 | 5 | *Cpe* |  | | | |
| Q3UC65 | Arginine/serine-rich protein 1 | < 0.001 | -1.62 | 5 | 5 | *Rsrp1* |  | | | |
| Q9EPR5 | VPS10 domain-containing receptor SorCS2 | < 0.0001 | -1.65 | 5 | 5 | *Sorcs2* |  | | | |
| P06797 | Cathepsin L1 | < 0.05 | -1.65 | 5 | 5 | *Ctsl* |  | | | |
| Q80W65 | Proprotein convertase subtilisin/kexin type 9 | < 0.01 | -1.66 | 5 | 5 | *Pcsk9* |  | | | |
| P18242 | Cathepsin D | < 0.05 | -1.70 | 5 | 5 | *Ctsd* |  | | | |
| Q60870 | Receptor expression-enhancing protein 5 | < 0.05 | -1.70 | 5 | 5 | *Reep5* |  | | | |
| Q8CGC6 | RNA-binding protein 28 | < 0.05 | -1.72 | 5 | 5 | *Rbm28* |  | | | |
| Q03137 | Ephrin type-A receptor 4 | < 0.05 | -1.82 | 5 | 4 | *Epha4* |  | | | |
| Q78J03 | Methionine-R-sulfoxide reductase B2, mitochondrial | < 0.01 | -1.85 | 5 | 5 | *Msrb2* |  | | | |
| P97857 | A disintegrin and metalloproteinase with thrombospondin motifs 1 | < 0.001 | -1.86 | 5 | 5 | *Adamts1* |  | | | |
| Q9R001 | A disintegrin and metalloproteinase with thrombospondin motifs 5 | < 0.01 | -1.87 | 5 | 5 | *Adamts5* |  | | | |
| P70180 | Atrial natriuretic peptide receptor 3 | < 0.05 | -1.88 | 5 | 5 | *Npr3* |  | | | |
| P05202 | Aspartate aminotransferase, mitochondrial | < 0.05 | -1.90 | 5 | 5 | *Got2* |  | | | |
| Q62351 | Transferrin receptor protein 1 | < 0.00001 | -2.16 | 5 | 5 | *Tfrc* |  |  |  |  |
| Q3UGP9 | Leucine-rich repeat-containing protein 58 | < 0.0001 | -2.23 | 5 | 5 | *Lrrc58* |  | | | |
| Q811J3 | Iron-responsive element-binding protein 2 | < 0.01 | -4.50 | 5 | 3 | *Ireb2* |  | | | |

***Supplementary Table 4: Differentially expression proteins in mesangial cells following oxDHA+AO stimulation, based on the analysis of the cellular proteome.***

*This table lists the proteins, which are differentially expressed between the oxDHA+AO-stimulated cells and the control group, including their respective protein ID, name, p-value, fold-change, and gene name. The column “valid values” indicated the number of valid measurements for each protein, while only proteins with at least 3 valid measurements in both groups were included. The column “pathways” shows the relevant pathways associated with each protein, color-coded to match pathway categories presented in related analysis tables (Table 1). Proteins are sorted in descending order by their fold-change values. Only statistically significant proteins (p < 0.05) with a fold-change ≥ 1.5 or ≤ -1.5 relative to the control were included. Albumin was excluded, due to high albumin concentration in the stimulation media. Color coded: ferroptosis, red; fatty acid metabolism, blueish; fatty acid degradation, green; PPAR signaling pathway, yellow. *, proteins highly involved in the respective pathway, but are not identified through STRING analysis.*


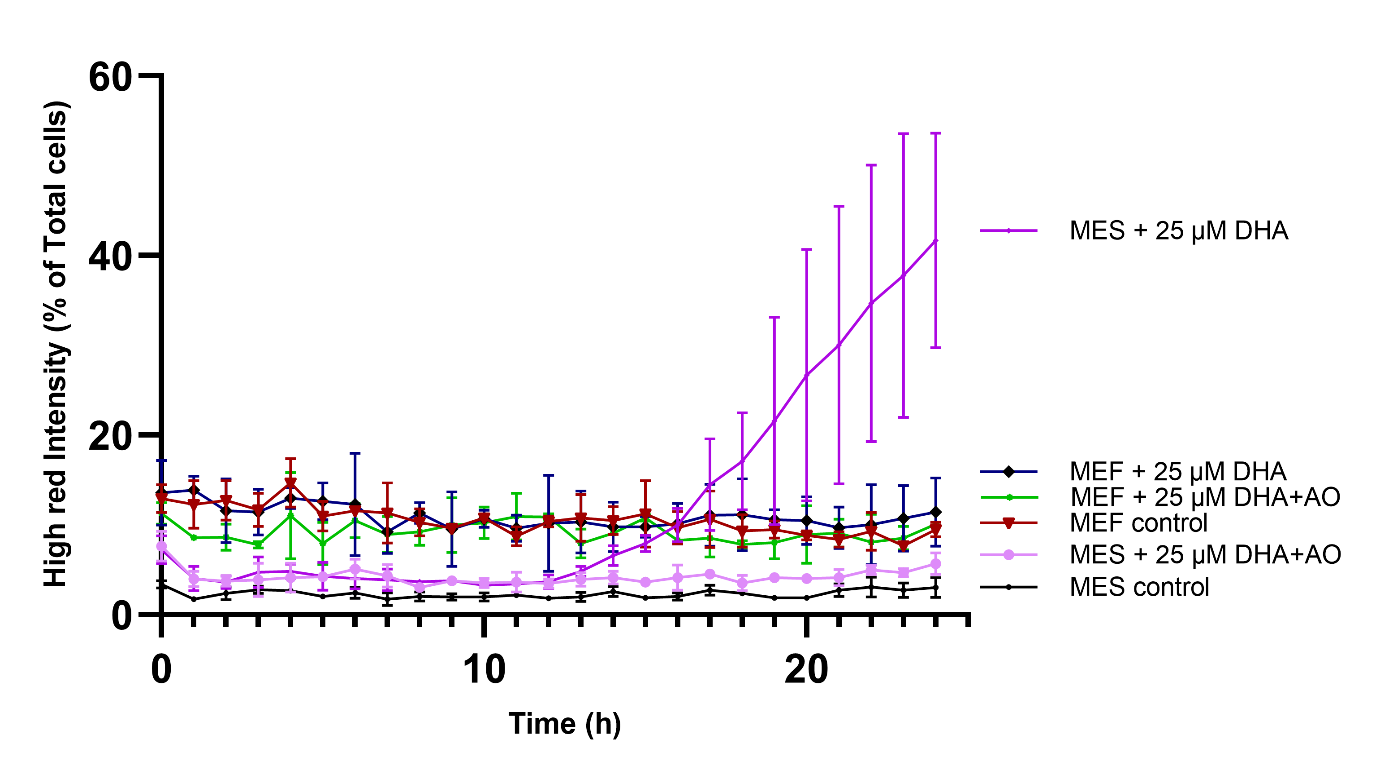


**Supplementary Figure 1:** **Preliminary testing of the impact of DHA and antioxidants on mouse embryonic fibroblasts (MEF) and murine mesangial cells (MES) on cell viability**. Quantification of cell death, based on red staining of dead cells (high red intensity). MEF’s and MES were stimulated with control media, 25 µM DHA, or 25 µM DHA+AO. Experiment performed with the Incucyte. Results are depicted as means (n=2, biological replicates) ± SD. Each biological replicate was measured in technical duplicates.


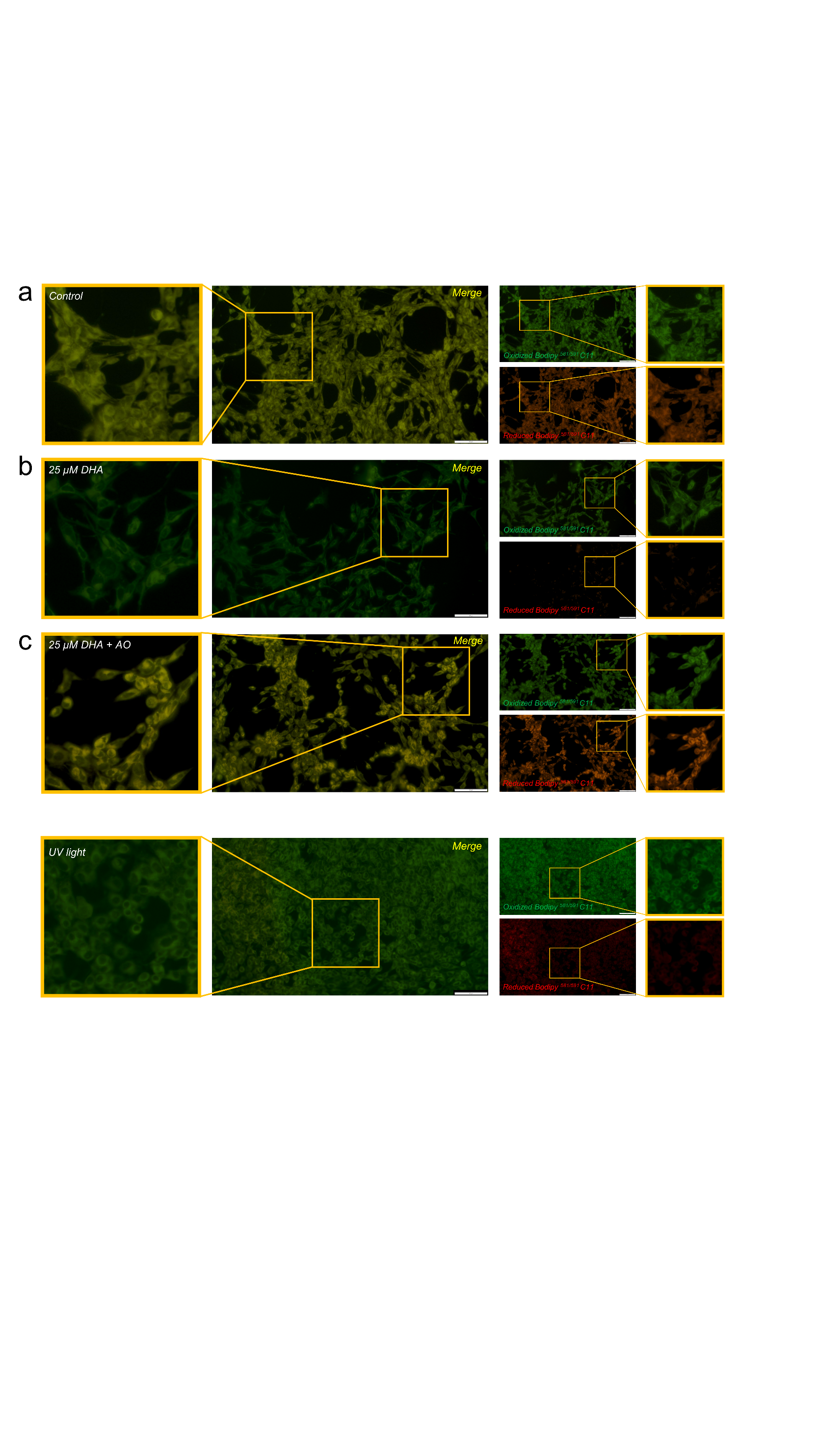


**Supplementary Figure 2:** **UV light-induced lipid peroxidation detected by Bodipy^581/591^ C‑11 staining.** Representative fluorescence microscopy images of cells stained with the lipid peroxidation sensor Bodipy^581/591^ C‑11 after UV light exposure for 5 minutes as a positive control for lipid peroxidation. The merged overlay (left) and individual oxidized (green, right, top) and reduced (red, right, bottom) channels are shown. For better visualization of the individual channels, brightness was uniformly increased (oxidized signal: +15%; reduced signal: +30%) without contrast adjustment. Merged images represent unmodified overlays. Scale bar: 100 µm.
