# Supplementary material for: Antioxidants rescue murine mesangial cells from docosahexaenoic acid-induced ferroptosis
Source: Mol Cell Pediatr. 2026 Jan 5;13:2. doi: 10.1186/s40348-025-00215-y (PMC12770095; doi:10.1186/s40348-025-00215-y)
Supplement: Supplementary file 2 — Supplementary Material 2. [file 40348_2025_215_MOESM2_ESM.docx]

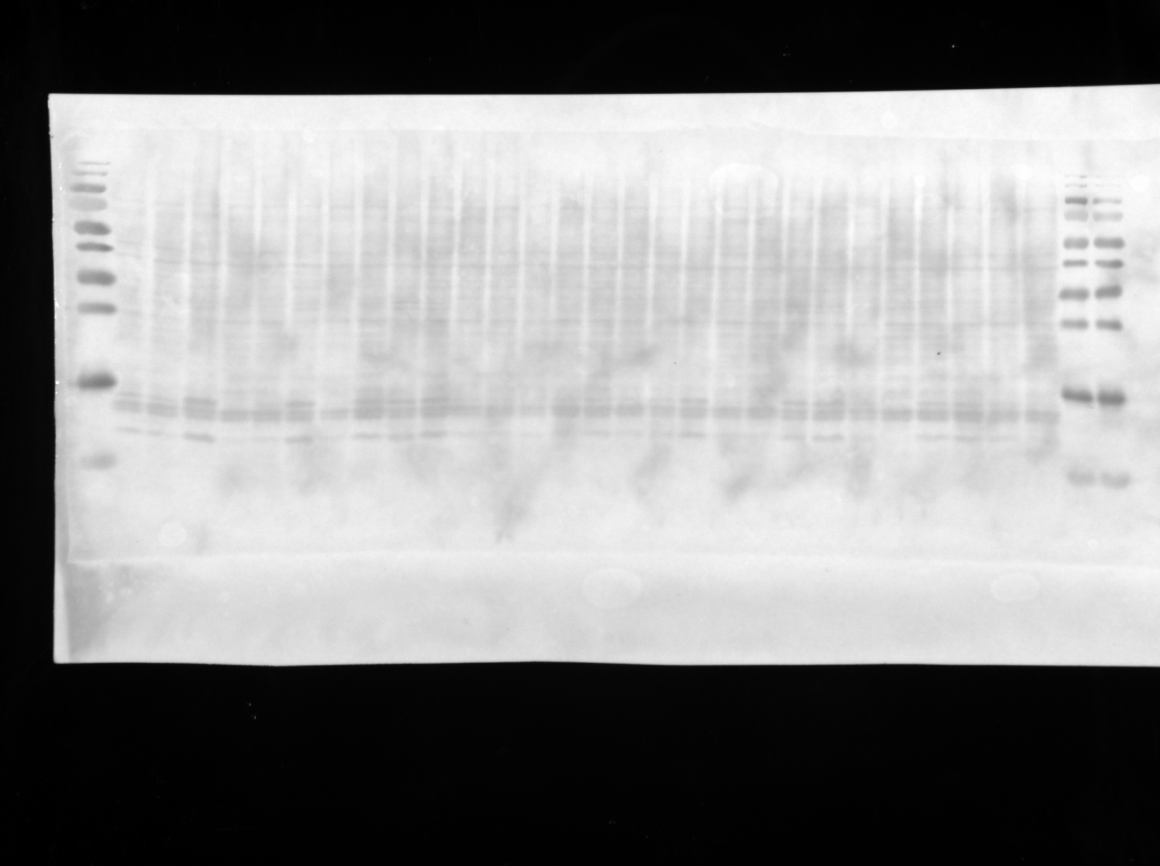


**Supplementary Figure 3a: Ponceau S staining of Western blot membrane used for caspase-3 and cleaved caspase-3 detection (Blot A).** Ponceau S staining of the transfer membrane, confirming successful protein transfer. Lanes are organized as follows: 1-4, control; 5-8, Carrier; 9-12, 25 µM DHA; 13-16, 25 µM DHA+AO; 17-20, AO; 21-24, 25 µM oxDHA; 25-28, 25 µM oxDHA+AO. Each group comprises four biological replicates (n=4). Membrane was cut afterwards for caspase-3 and cleaved caspase-3 detection (Blot A).


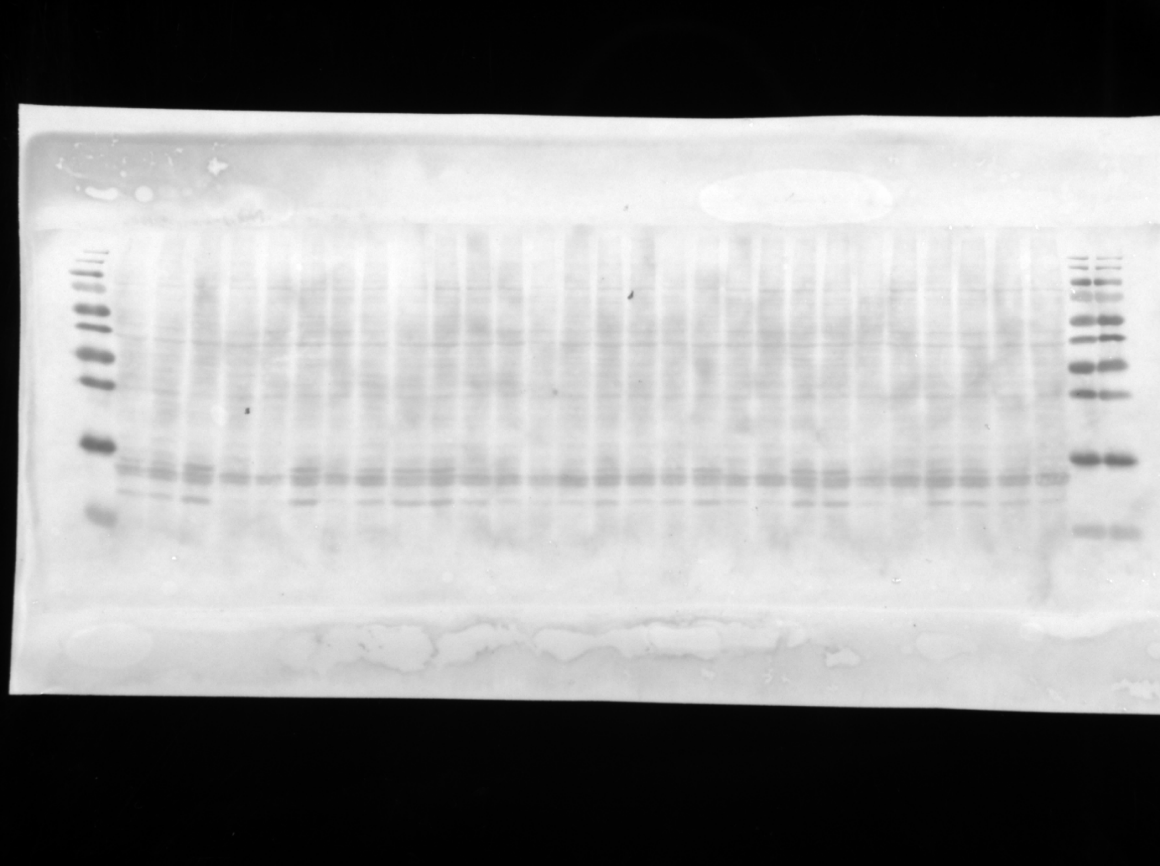


**Supplementary Figure 3b:** **Ponceau S staining of Western blot membrane used for GPX4 and FTH1 detection (Blot B).** Ponceau S staining of the transfer membrane, confirming successful protein transfer. Lanes are organized as follows: 1-4, control; 5-8, Carrier; 9-12, 25 µM DHA; 13-16, 25 µM DHA+AO; 17-20, AO; 21-24, 25 µM oxDHA; 25-28, 25 µM oxDHA+AO. Each group comprises four biological replicates (n=4). Membrane was cut afterwards for GPX4 and FTH1 detection (Blot B).


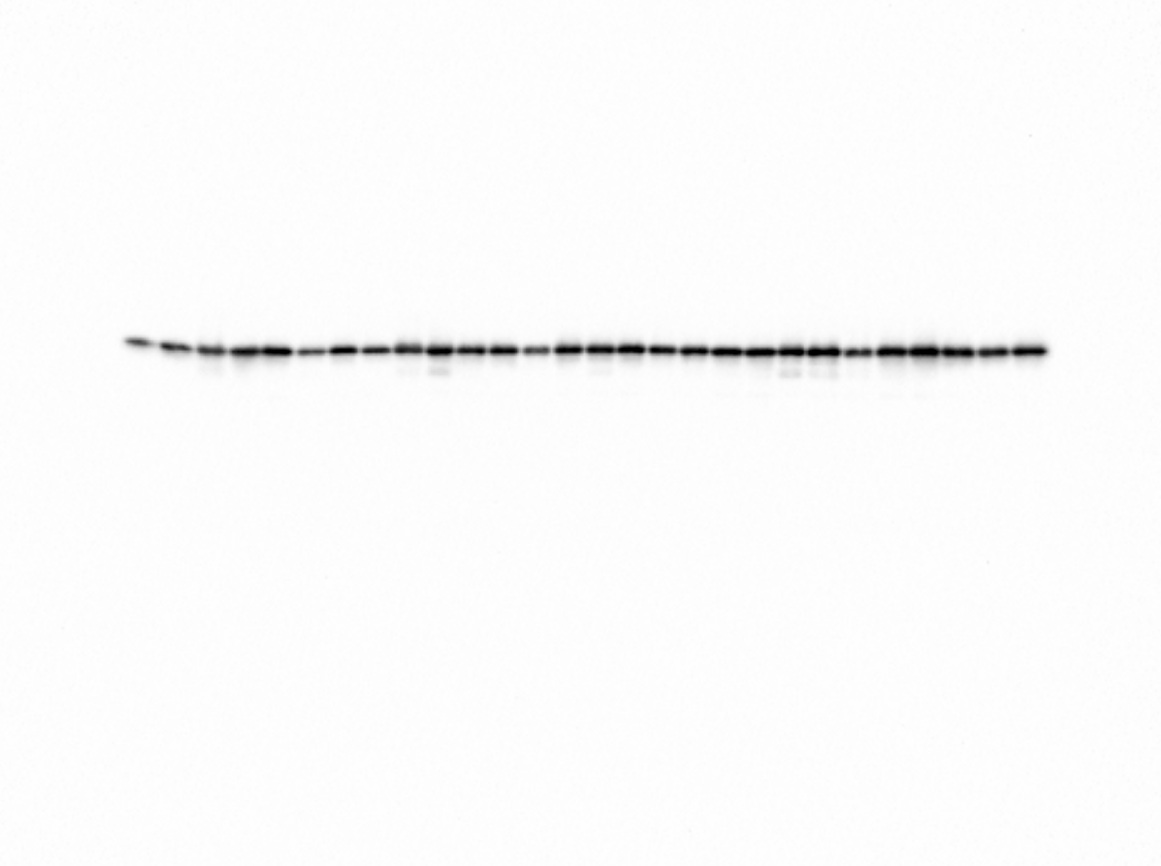


**Supplementary Figure 4a: Full, uncropped Western Blot membrane showing caspase-3 detection**. Representative full-length Western Blot membranes corresponding to Figure 3d, showing total caspase-3 protein levels across all experimental conditions. Lanes are organized as follows: 1-4, control; 5-8, Carrier; 9-12, 25 µM DHA; 13-16, 25 µM DHA+AO; 17-20, AO; 21-24, 25 µM oxDHA; 25-28, 25 µM oxDHA+AO. Each group comprises four biological replicates (n=4).


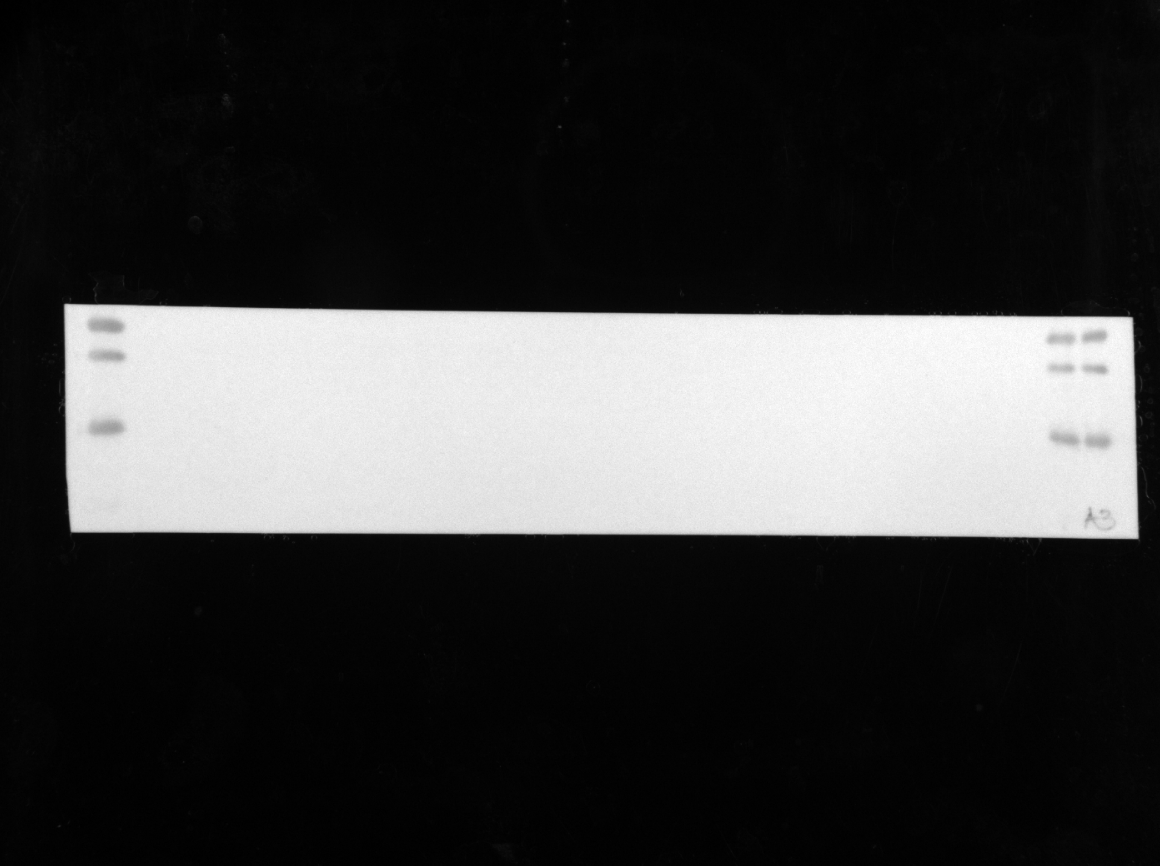


**Supplementary Figure 4b: Full, uncropped Western Blot membrane with protein marker for caspase-3 detection.** PageRuler Prestained Protein Ladder, 10-170 kDa; bands represent here 35, 25, and 15 kDa (top to bottom) used for caspase-3 Western blot. The molecular weight marker shown is specific to this blot.


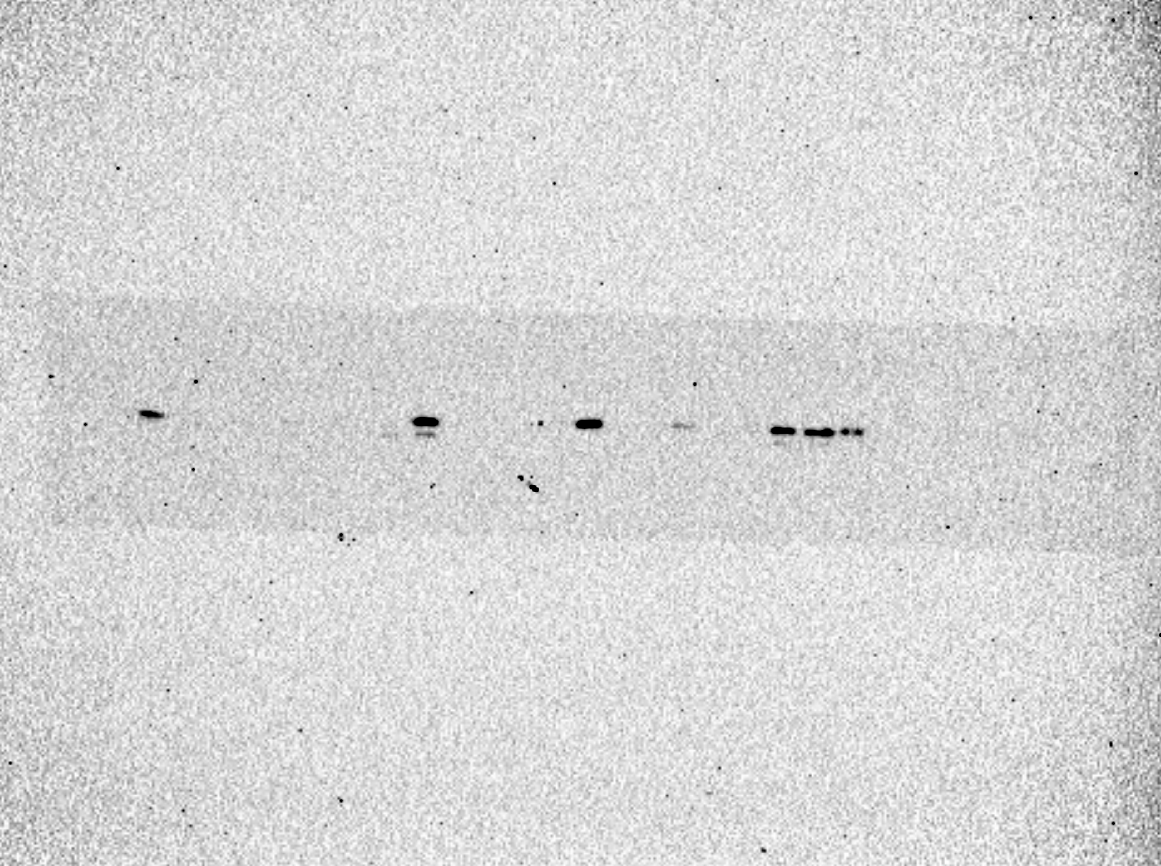


**Supplementary Figure 5a: Full, uncropped Western Blot membrane showing cleaved caspase-3 detection**. Representative full-length Western Blot membranes corresponding to Figure 3d, showing total cleaved caspase-3 protein levels across all experimental conditions. Lanes are organized as follows: 1-4, control; 5-8, Carrier; 9-12, 25 µM DHA; 13-16, 25 µM DHA+AO; 17-20, AO; 21-24, 25 µM oxDHA; 25-28, 25 µM oxDHA+AO. Each group comprises four biological replicates (n=4).


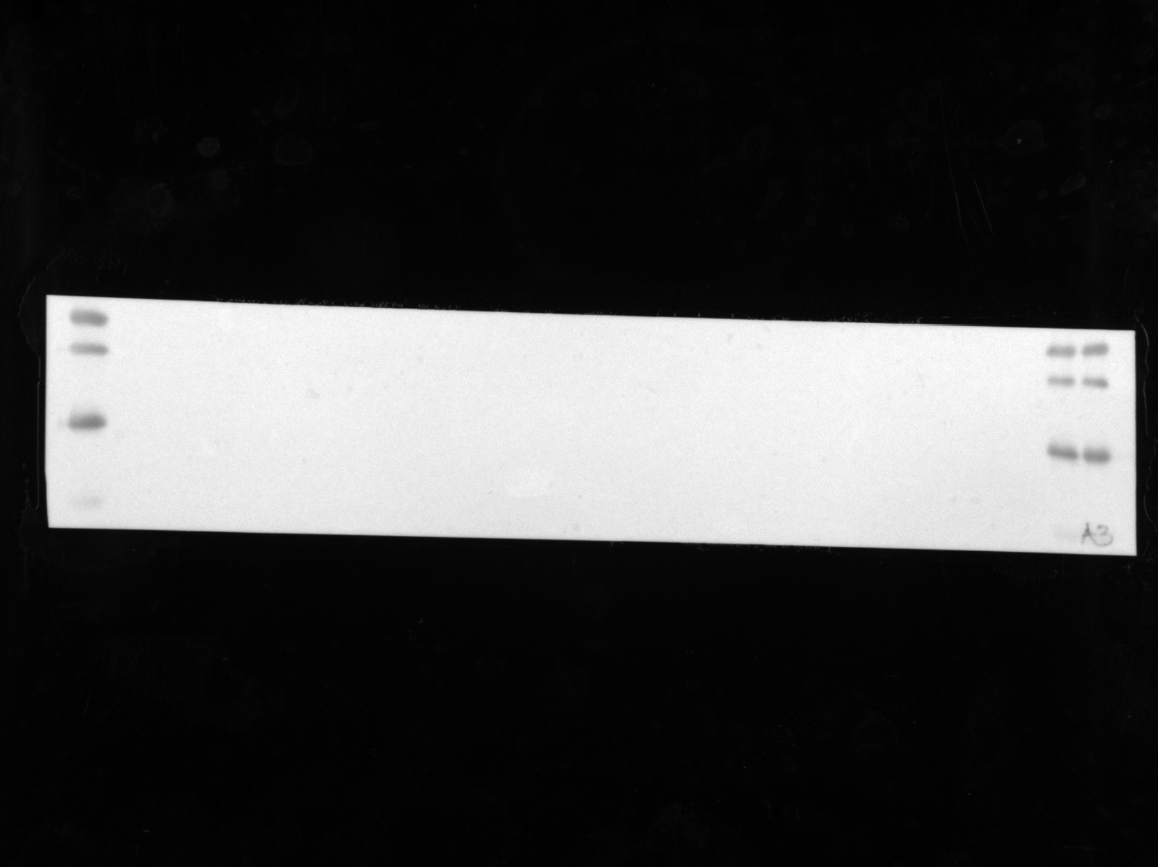


**Supplementary Figure 5b: Full, uncropped Western Blot membrane with protein marker for cleaved caspase-3 detection.** PageRuler Prestained Protein Ladder, 10-170 kDa; bands represent here 35, 25, and 15 kDa (top to bottom) used for cleaved caspase-3 Western blot. The molecular weight marker shown is specific to this blot.


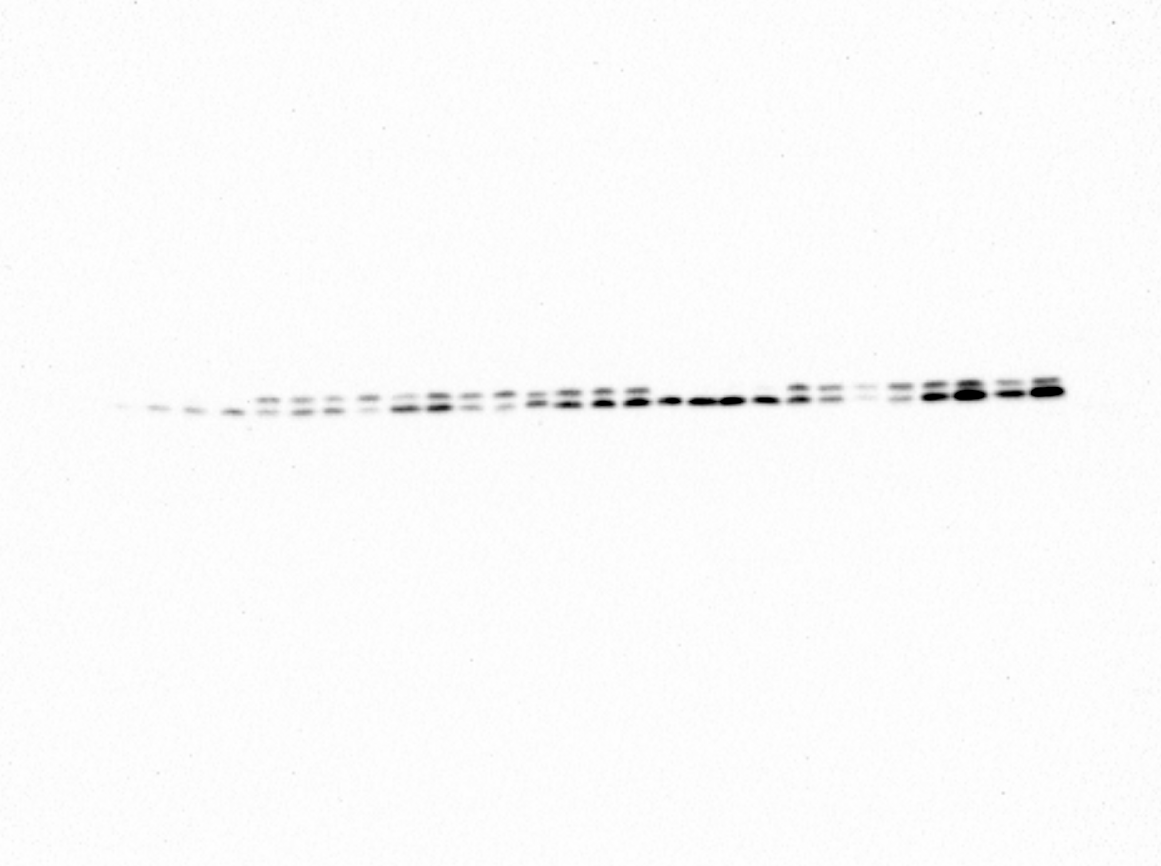


**Supplementary Figure 6a: Full, uncropped Western Blot membrane showing GPX4 detection**. Representative full-length Western Blot membranes corresponding to Figure 3a, showing total GPX4 protein levels across all experimental conditions. Lanes are organized as follows: 1-4, control; 5-8, Carrier; 9-12, 25 µM DHA; 13-16, 25 µM DHA+AO; 17-20, AO; 21-24, 25 µM oxDHA; 25-28, 25 µM oxDHA+AO. Each group comprises four biological replicates (n=4).


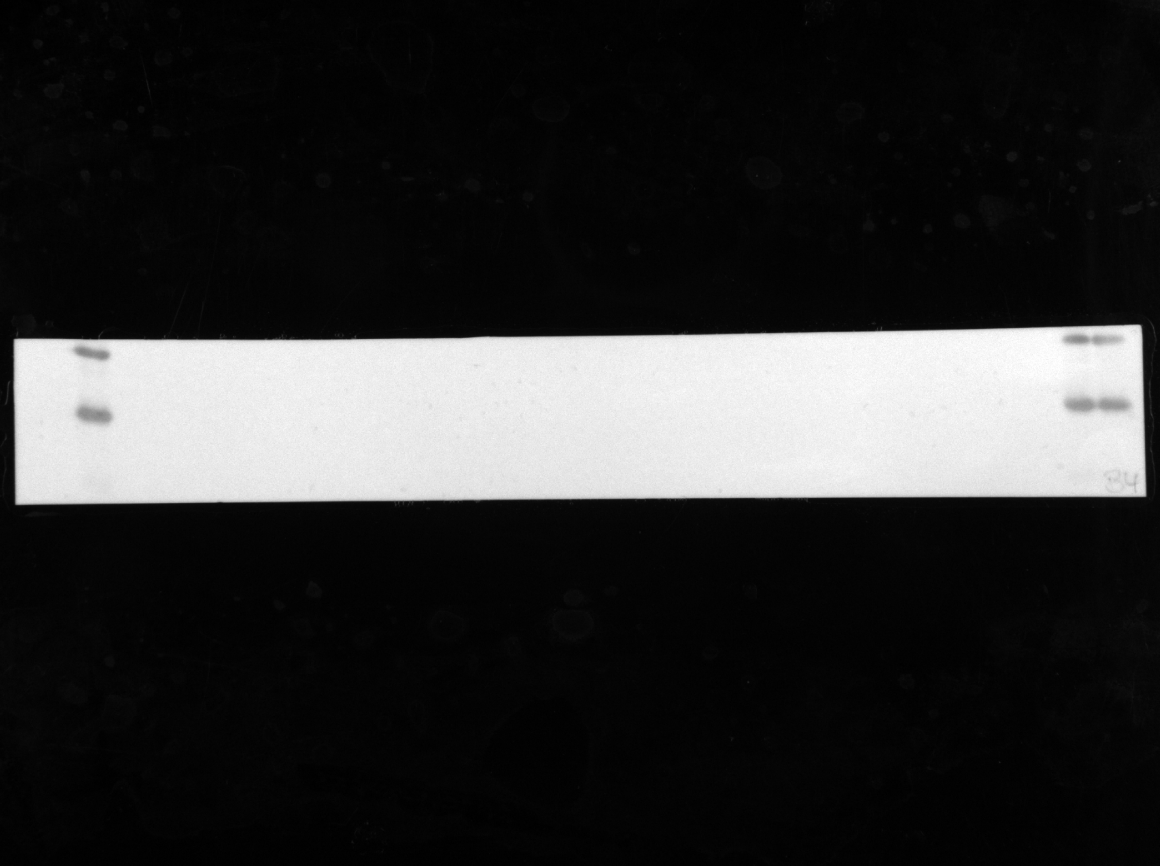


**Supplementary Figure 6b: Full, uncropped Western Blot membrane with protein marker for GPX4 detection.** PageRuler Prestained Protein Ladder, 10-170 kDa; bands represent here 25 and 15 kDa (top to bottom) used for GPX4 Western blot. The molecular weight marker shown is specific to this blot.


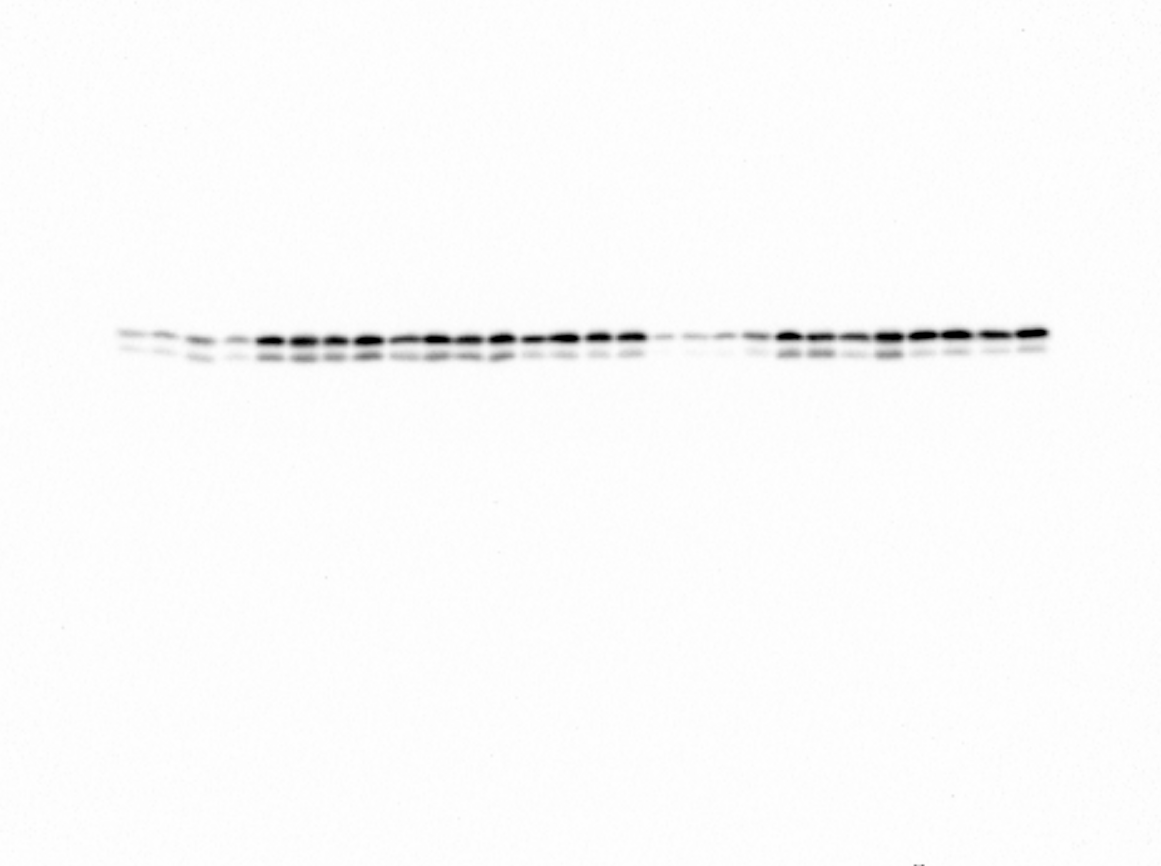


**Supplementary Figure 7a:** **Full, uncropped Western Blot membrane showing FTH1 detection**. Representative full-length Western Blot membranes corresponding to Figure 3a, showing total FTH1 protein levels across all experimental conditions. Lanes are organized as follows: 1-4, control; 5-8, Carrier; 9-12, 25 µM DHA; 13-16, 25 µM DHA+AO; 17-20, AO; 21-24, 25 µM oxDHA; 25-28, 25 µM oxDHA+AO. Each group comprises four biological replicates (n=4).


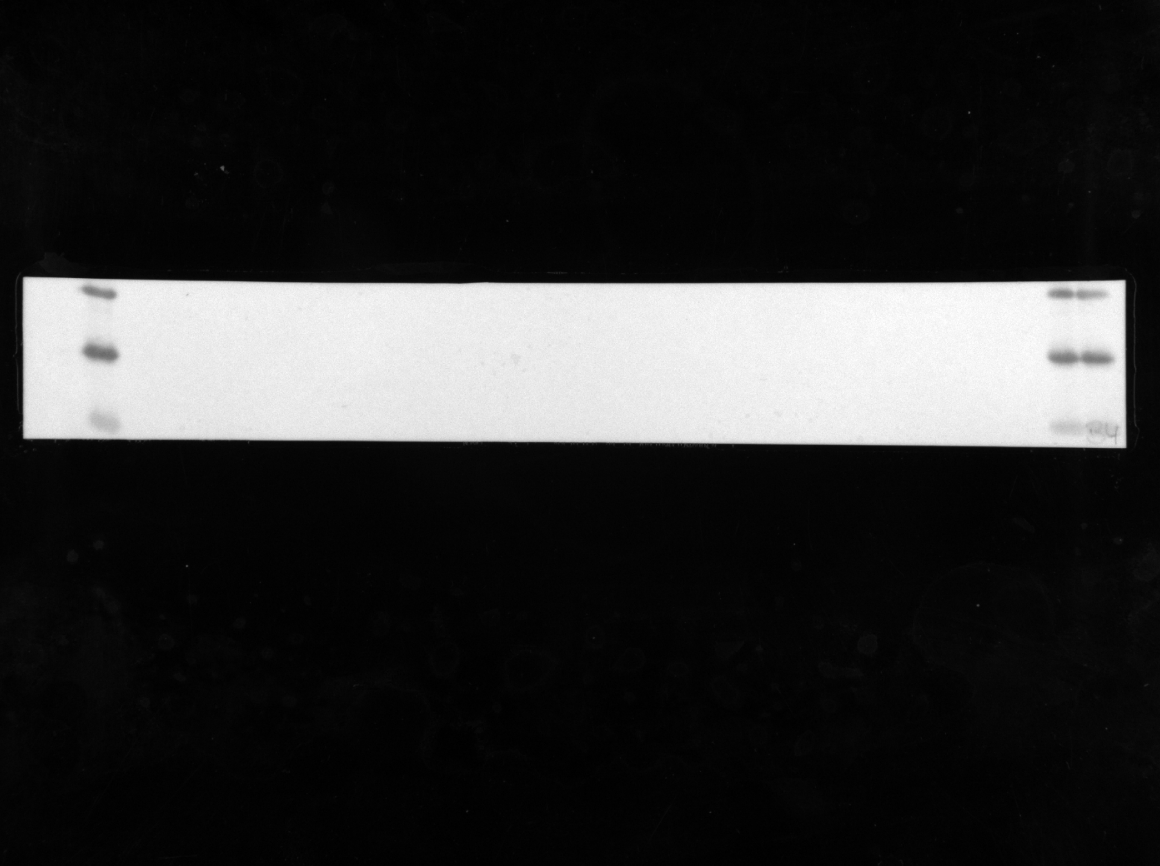


**Supplementary Figure 7b: Full, uncropped Western Blot membrane with protein marker for FTH1 detection.** PageRuler Prestained Protein Ladder, 10-170 kDa; bands represent here 25, 15, and 10 kDa (top to bottom) used for FTH1 Western blot. The molecular weight marker shown is specific to this blot.


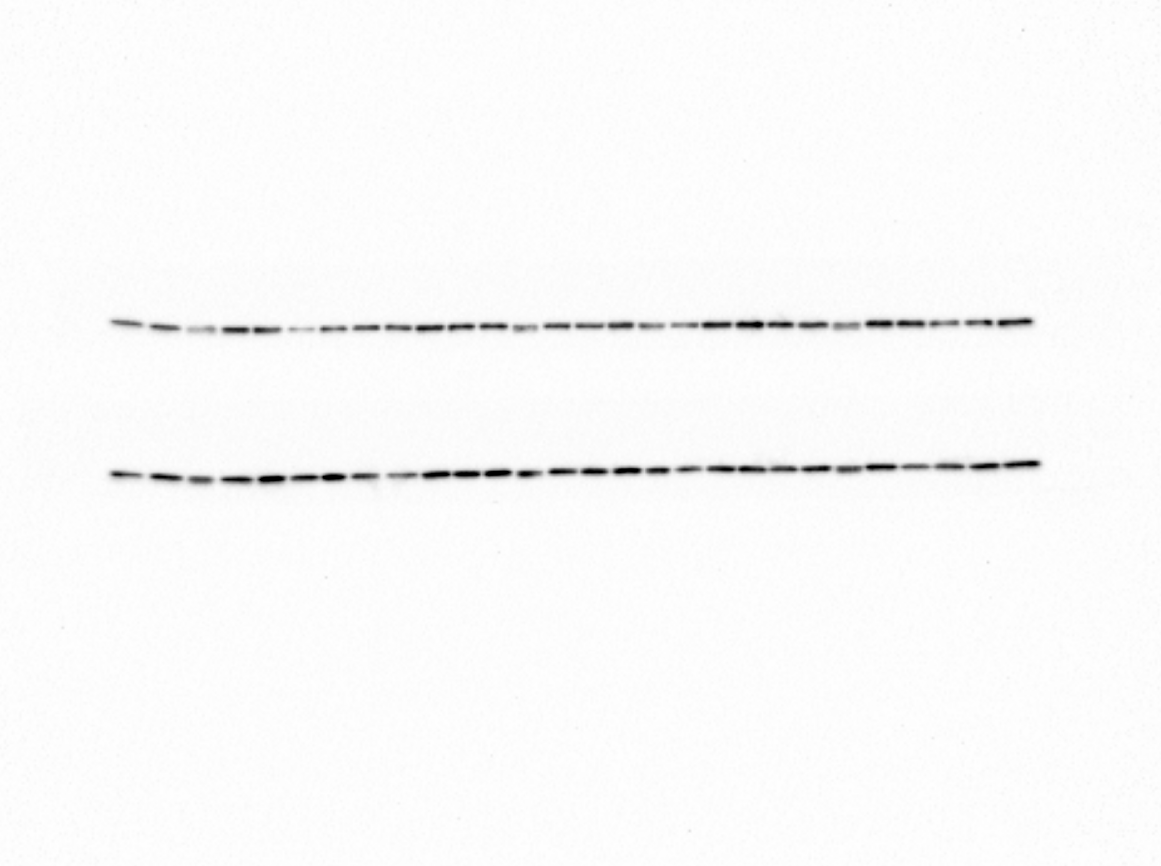


**Supplementary Figure 8a: Full, uncropped Western Blot membrane showing α-Tubulin detection**. Representative full-length Western Blot membranes corresponding to Figure 3a, d, showing total α-Tubulin protein levels across all experimental conditions. Lanes are organized as follows: 1-4, control; 5-8, Carrier; 9-12, 25 µM DHA; 13-16, 25 µM DHA+AO; 17-20, AO; 21-24, 25 µM oxDHA; 25-28, 25 µM oxDHA+AO. Each group comprises four biological replicates (n=4). Upper lane represents blot A (Caspase-3, cleaved Caspase-3), lower lane represents Blot B (GPX4, FTH1).


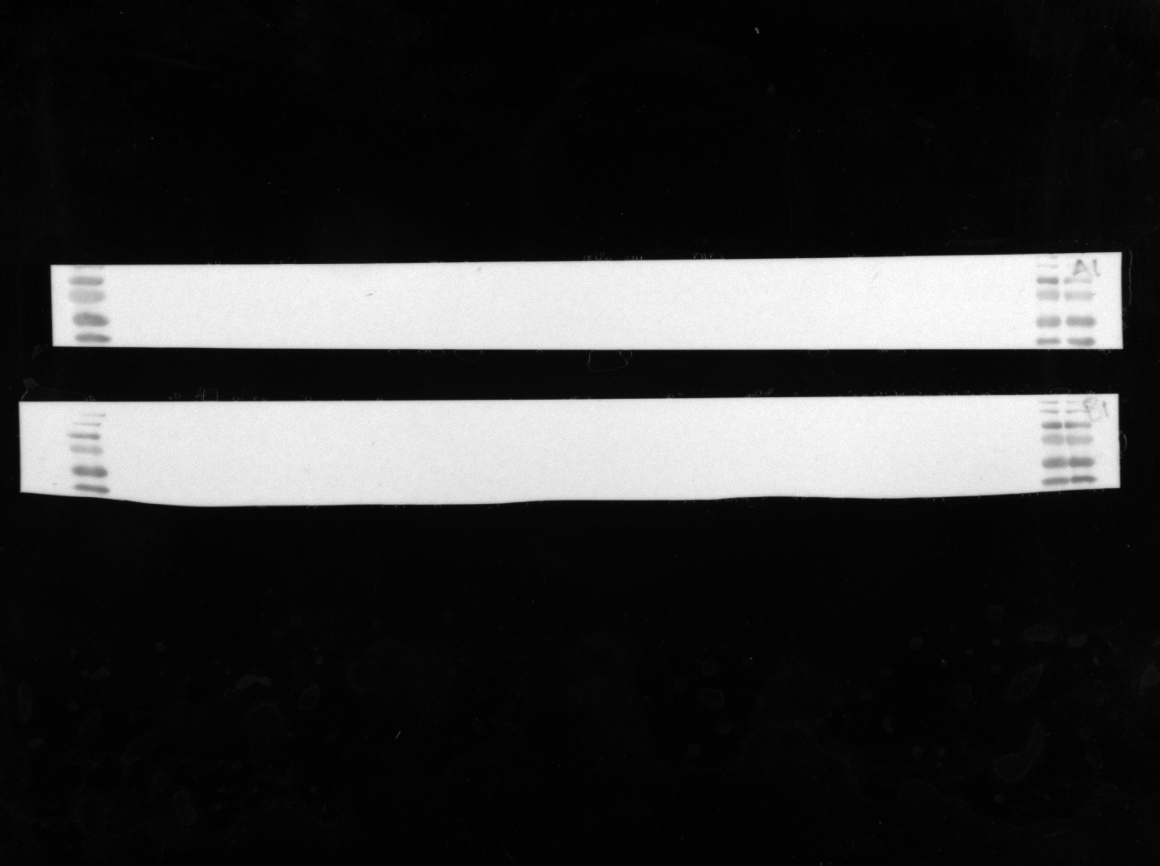


**Supplementary Figure 8b: Full, uncropped Western Blot membrane with protein marker for α-Tubulin detection.** PageRuler Prestained Protein Ladder, 10-170 kDa; bands represent here 100, 70, 55 and 40 kDa (top to bottom) used for α-Tubulin Western blot. The molecular weight marker shown is specific to this blot. Upper represents Blot A, lower represents Blot B.
